# TRIM29 controls enteric RNA virus-induced intestinal inflammation by targeting NLRP6 and NLRP9b signaling pathways

**DOI:** 10.1016/j.mucimm.2024.10.004

**Published:** 2024-10-11

**Authors:** Junying Wang, Ling Wang, Wenting Lu, Naser Farhataziz, Anastasia Gonzalez, Junji Xing, Zhiqiang Zhang

**Affiliations:** a Immunobiology and Transplant Science Center, Department of Surgery, Houston Methodist Academic Institute, Houston Methodist, Houston, TX 77030, USA; b Department of Obstetrics and Gynecology, The Second Hospital of Jilin University, Changchun, 130021, China; c Department of Cardiovascular Sciences, Houston Methodist Academic Institute, Houston Methodist, Houston, TX 77030, USA; d Department of Surgery, Weill Cornell Medicine, Cornell University, New York, NY 10065, USA

## Abstract

Infections by enteric virus and intestinal inflammation are recognized as a leading cause of deadly gastroenteritis, and NLRP6 and NLRP9b signaling control these infection and inflammation. However, the regulatory mechanisms of the NLRP6 and NLRP9b signaling in enteric viral infection remain unexplored. In this study, we found that the E3 ligase TRIM29 suppressed type III interferon (IFN-λ) and interleukin-18 (IL-18) production by intestinal epithelial cells (IECs) when exposed to polyinosinic:polycytidylic acid (poly I:C) and enteric RNA viruses. Knockout of TRIM29 in IECs was efficient to restrict intestinal inflammation triggered by the enteric RNA viruses, rotavirus in suckling mice, and the encephalomyocarditis virus (EMCV) in adults. This attenuation in inflammation was attributed to the increased production of IFN-λ and IL-18 in the IECs and more recruitment of intraepithelial protective Ly6A^+^CCR9^+^CD4^+^ T cells in small intestines from TRIM29-deficient mice. Mechanistically, TRIM29 promoted K48-linked ubiquitination, leading to the degradation of NLRP6 and NLRP9b, resulting in decreased IFN-λ and IL-18 secretion by IECs. Our findings reveal that enteric viruses utilize TRIM29 to inhibit IFN-λ and inflammasome activation in IECs, thereby facilitating viral-induced intestinal inflammation. This indicates that targeting TRIM29 could offer a promising therapeutic strategy for alleviating gut diseases.

Gastroenteritis causes significant morbidity and mortality worldwide and represents one of major socioeconomic burdens^1,2^. As an inflammatory condition of the stomach and intestines, it manifests through tissue inflammation, epithelial barrier disruption, malabsorption, and diarrhea^1^. Enteric viruses, characterized by fecal-oral transmission and gastrointestinal replication, are recognized as a leading cause of deadly gastroenteritis worldwide, particularly among children in developing countries^3^. Pathogenic enteric viruses, causing viral gastroenteritis in human, are mainly RNA viruses, including rotavirus, norovirus, enterovirus, reovirus, and astrovirus^4,5^. Adding a layer of complexity to the current landscape is the Severe Acute Respiratory Syndrome Coronavirus 2 (SARS-CoV-2), responsible for the ongoing coronavirus disease 2019 (COVID-19) pandemic. Recent studies indicate its potential to cause intestinal infections, as evidenced both *in vitro* ^6,7^ and *in vivo* in animal models^8,9,10^. However, definitive proof of gastrointestinal damage directly attributable to SARS-CoV-2 remains elusive^3^. Furthermore, the mechanisms causing diarrhea in COVID-19 patients are not yet fully understood. In light of the continuing challenges posed by the COVID-19 pandemic, it is imperative to deepen our understanding of viral gastroenteritis pathogenesis, paving the way for effective therapeutic interventions and better vaccines.

Intestinal epithelial cells (IECs) line the intestinal tract and serve as a first line of defense against enteric viruses^4^. IECs possess specialized RNA virus sensors that detect invading enteric RNA viruses, initiating an antiviral innate immune response through the production of type I interferon (IFN-I) and type III interferon (IFN lambdas, IFN-λs)^11,12,13–15^. Both IFN-I and IFN-λs activate antiviral mechanisms in virus-infected and uninfected bystander cells and synergistically steer the maturation of adaptive immune responses against enteric viruses^16,17^. Recent studies, including our own, have spotlighted the RNA helicase DHX15 as an RNA sensor for various enteric RNA viruses, such as rotavirus, reovirus, encephalomyocarditis virus (EMCV) and norovirus^18,19^. On detecting the double-stranded RNA (dsRNA) of these viruses, DHX15 mobilizes NLRP6 and MAVS, culminating in the production of IFN-I, IFN-λs, and interferon-stimulated genes (ISGs) in IECs, which then act to restrict these enteric RNA viruses^18,19,20^. In addition, inflammasome plays a pivotal role in host defense by recognizing viral infection and triggering responses from the innate immune system ^21,22,23^. IECs are fortified with various RNA virus inflammasome receptors that sense and respond to invading enteric viruses, chiefly by activating the inflammasome and thereby triggering the release of the cytokine IL-18^24,25^. Our research identified DHX15 as the sensor for these viruses, facilitating the activation of the NLRP6 inflammasome and the subsequent production of IL-18, essential for managing infections from viruses like rotavirus and reovirus^18^. Furthermore, another RNA helicase, DHX9, is believed to detect dsRNA of rotavirus, activating the NLRP9b inflammasome, which restricts rotavirus infection in IECs *in vivo*^26^. Intriguingly, only NLRP6 and NLRP9b, not other members of the NLR family like NLRP3, have been identified as being selectively expressed in mouse IECs^26^. Despite extensive research into the regulatory mechanisms of other NLRP family proteins in inflammasome activation, studies focused on NLRP6 and NLRP9b remain scant. Given their uniquely high expression in IECs, it’s crucial to delve deeper into the regulatory dynamics of NLRP6 and NLRP9b inflammasomes, especially in the context of enteric virus infections and associated intestinal ailments. Recently, the newly recruited intraepithelial Ly6A^+^CCR9^+^CD4^+^ T cells have been shown to protect against enteric adenovirus infection^27^. However, how enteric viruses alter IECs and how virus-induced epithelial cell changes impact the function and acquisition of innate-like properties by the intraepithelial Ly6A^+^CCR9^+^CD4^+^ T cells are unknown.

We have previously shown that TRIM29 is instrumental in the host defense against respiratory threats such as influenza virus, bacteria Haemophilus influenzae, and oncogenic Epstein-Barr virus in respiratory tract by negatively regulating innate immune responses in alveolar macrophages^28^ and airway epithelial cells^29^. However, the role of TRIM29 in modulating mucosal immunity within the intestines, particularly during viral gastroenteritis, remains unknown. In this study, we generated IEC-specific *TRIM29*-knockout mice to investigate the functions of TRIM29 in IECs during viral gastroenteritis. We found that TRIM29 targeted NLRP6 and NLRP9b for ubiquitination-mediated degradation to suppress IFN-λ3 and inflammasome-derived cytokine IL-18 production in human and mouse IECs following double strand RNA (dsRNA) poly I:C stimulation or enteric RNA virus infection. IEC-specific TRIM29 ablation mitigated intestinal inflammation caused by enteric RNA viruses *in vivo* duo to increased IFN-λ3 and IL-18 production and an augmented recruitment of protective intraepithelial Ly6A^+^CCR9^+^CD4^+^ T cells in small intestines. Thus, our studies reveal the viral role of TRIM29 in orchestrating innate immune reactions to enteric RNA viruses in IECs and in moderating intestinal inflammation triggered by these viruses.

## Results

### TRIM29 inhibits IFN-λ3 and IL-18 production in human HT-29 IECs following poly I:C stimulation or infection by enteric RNA virus

**Fig. 1.**
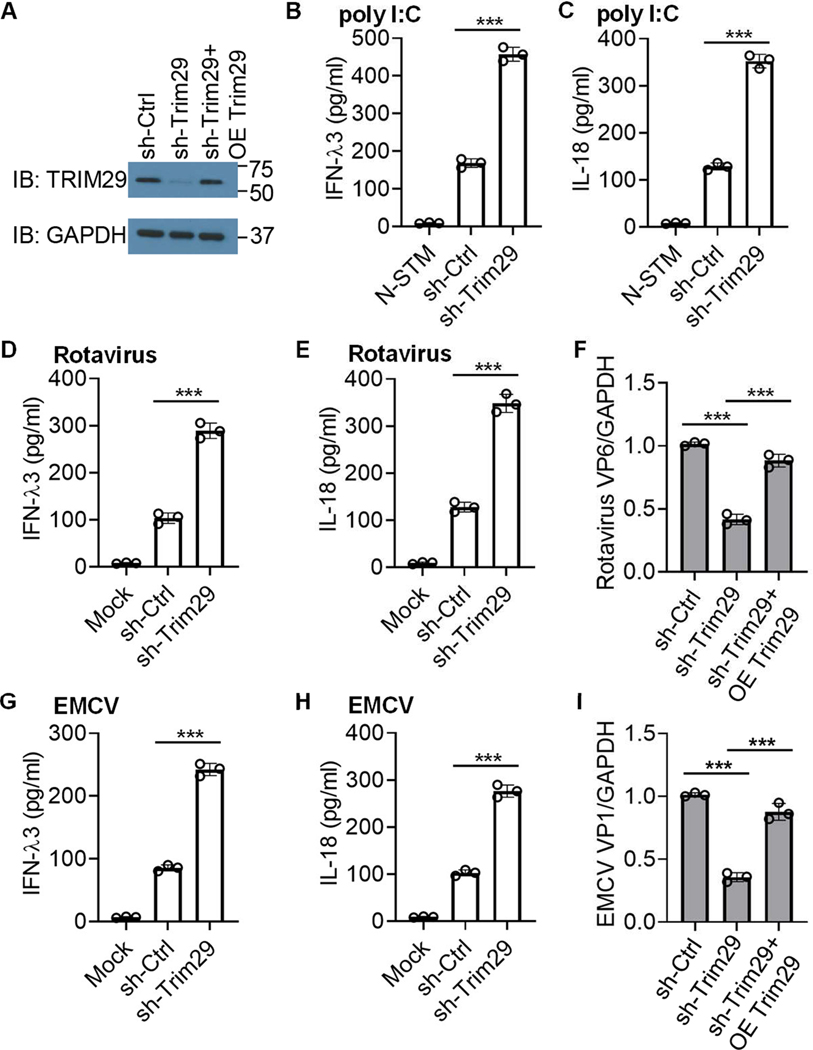
TRIM29 inhibits IFN-λ3 and IL-18 production in human HT-29 IECs following poly I:C stimulation and enteric RNA virus infection. (**A**) Immunoblot (IB) showing the knockdown efficiency of shRNAs targeting the Trim29 gene (sh-Trim29) in HT-29 IECs and overexpression efficiency of TRIM29 (OE Trim29) in HT-29 IECs with sh-Trim29 treatment followed by transfection of HA-TRIM29 plasmid by Lipofectamine 3000. Nontargeting shRNA served as a control (sh-Ctrl). GAPDH blots are shown as loading controls. The position of protein markers (shown in kDa) is indicated on the right. (**B-C**) ELISA of IFN-λ3 (**B**) and IL-18 (**C**) production from human HT-29 IECs with the indicated shRNA after a 20 h stimulation with 5 μg/ml poly I:C delivered by Lipofectamine 3000. N-STM, scrambled shRNA-treated HT-29 IECs without stimulation. (**D-E,G-H**) ELISA of IFN-λ3 (**D, G**) and IL-18 (**E, H**) production from human HT-29 IECs with the indicated shRNA after a 20 h infection with enteric RNA viruses including simian rotavirus SA-11 strain (**D-E**) and EMCV K strain (**G-H**) at a multiplicity of infection (MOI) of 5. Mock, scrambled shRNA(sh-Ctrl)-treated human HT-29 IECs without virus infection. (**F,I**) Quantification of expression of rotavirus VP6 gene (**F**) and EMCV VP1 gene (**I**) relative to GAPDH in human HT-29 IECs with sh-Ctrl, sh-Trim29 treatment or sh-Trim29 treatment followed by transfection of HA-TRIM29 plasmid after infection with rotavirus (**F**) or EMCV (**I**) at a MOI of 5. Data are represented as mean ± SD. ***P < 0.001 (unpaired *t* test). Data are representative of three technical replicates.

To investigate the biological roles of TRIM29 in IECs, we first established stable TRIM29-knockdown human HT-29 IECs through use of short hairpin RNA (shRNA). The Trim29-targeting shRNA produced efficient knockdown of TRIM29 expression (Fig. 1A). These cells were then stimulated by poly I:C (a mimic of RNA viruses) and the production of type III IFN IFN-λ3 and inflammasome cytokine IL-18 by the cultured HT-29 IECs was measured by ELISA. When contrasted with the control (sh-Ctrl) HT-29 IECs, there was a marked elevation in the production of cytokines IFN-λ3 (Fig. 1B) and IL-18 (Fig. 1C) in TRIM29-knockdown HT-29 IECs in response to poly I:C. These findings indicate that TRIM29 acts to suppress IFN-λ3 and IL-18 production in human HT-29 IECs when challenged with dsRNA poly I:C.

Enteric viruses, such as rotavirus and EMCV, could replicate in IECs and the influence of enteric viruses on intestine homeostasis and inflammation is just beginning to be unraveled ^1,19,30^. To ascertain the role of TRIM29 in regulating enteric RNA virus infection in human IECs, cytokines production was measured after culturing control (sh-Ctrl) and knockdown shRNA treated human HT-29 IECs followed by infection with two enteric RNA viruses including simian rotavirus SA-11 strain and EMCV K strain. TRIM29-knockdown HT-29 IECs produced more than 2-fold amount of IFN-λ3 (Fig. 1D and 1G) and IL-18 (Fig. 1Eand 1H) compared to the control (sh-Ctrl) HT-29 IECs upon infection with enteric RNA virus rotavirus (Fig. 1D and 1E) or EMCV (Fig. 1G and 1H), indicating TRIM29 inhibits IFN-λ3, and IL-18 production in human HT-29 IECs following enteric RNA virus infection. Importantly, knockdown of TRIM29in HT-29 IECs restricted the viral replication of enteric RNA virus rotavirus or EMCV, in comparison to the control (sh-Ctrl) IECs (Fig. 1F and 1I). Additionally, overexpression of TRIM29 in TRIM29 knock-down HT-29 IECs (Fig. 1A) rescued the viral replication of enteric RNA virus rotavirus or EMCV, in comparison to the TRIM29 knock-down (sh-Trim29) IECs (Fig. 1F and 1I). Therefore, these data strongly suggest that TRIM29 suppresses IFN-λ3 and IL-18 production in human HT-29 IECs when stimulated with poly I:C or infected by enteric virus.

### TRIM29 suppresses the production of IFN-λ3 and IL-18 in mouse IECs upon enteric RNA virus infection

We have found that TRIM29 suppresses IFN-λ3 and IL-18 production in human IECs upon exposure to dsRNA and enteric RNA viruses. This led us to investigate the role of TRIM29 in mouse IECs during enteric RNA virus infections. We isolated crypts from small intestine of both wild-type (*Trim29*^+/+^) and TRIM29 knockout (*Trim29*^*−/−*^) mice and cultured mouse intestinal organoids using crypts to get primary IECs, and subsequently infected these primary IECs from mouse intestinal organoids with two enteric RNA viruses including simian rotavirus SA-11 strain, which has been shown to replicate good in mouse intestinal organoids^31^, and EMCV followed by cytokine production detection using ELISA. Remarkably, primary IECs from mouse intestinal organoids of *Trim29*^*−/−*^ mice demonstrated substantially higher levels of IFN-λ3 and IL-18 compared to the cells from wild-type *Trim29*^+/+^ mice in response to enteric RNA virus rotavirus (Supplementary Fig. 1A and 1B) or EMCV (Supplementary Fig. 1C and 1D). Additionally, we observed a pronounced reduction in viral replication of both rotavirus (Supplementary Fig. 1E) and EMCV (Supplementary Fig. 1F) in the IECs from mouse intestinal organoids of *Trim29*^*−/−*^ mice compared to their *Trim29*^+/+^ counterparts. Next, we investigated the kinetics of IFN-λ3 and IL-18 production in primary IECs from mouse intestine organoids of both *Trim29*^+/+^ and *Trim29*^*−/−*^ mice at 10 h, 20 h, and 30 h after Rotavirus infection. We found that knockout of TRIM29 led to the peak production of IFN-λ3 and IL-18 at 20 h post-infection, with a subsequent decrease at 30 h after Rotavirus infection (Supplementary Fig. 1G and 1H). Additionally, mouse intestine macrophages of *Trim29*^*−/−*^ mice produced substantially higher levels of IFN-λ3 and IL-18 compared to the macrophages from wild-type *Trim29*^+/+^ mice in response to rotavirus (Supplementary Fig. 1I and 1J). These findings indicate that in the absence of TRIM29, there’s an enhancement in the production of IFN-λ3 and IL-18, which in turn restricts the replication of enteric RNA viruses in primary IECs from mouse intestinal organoids and intestine macrophages *in vitro*.

**Fig. 2.**
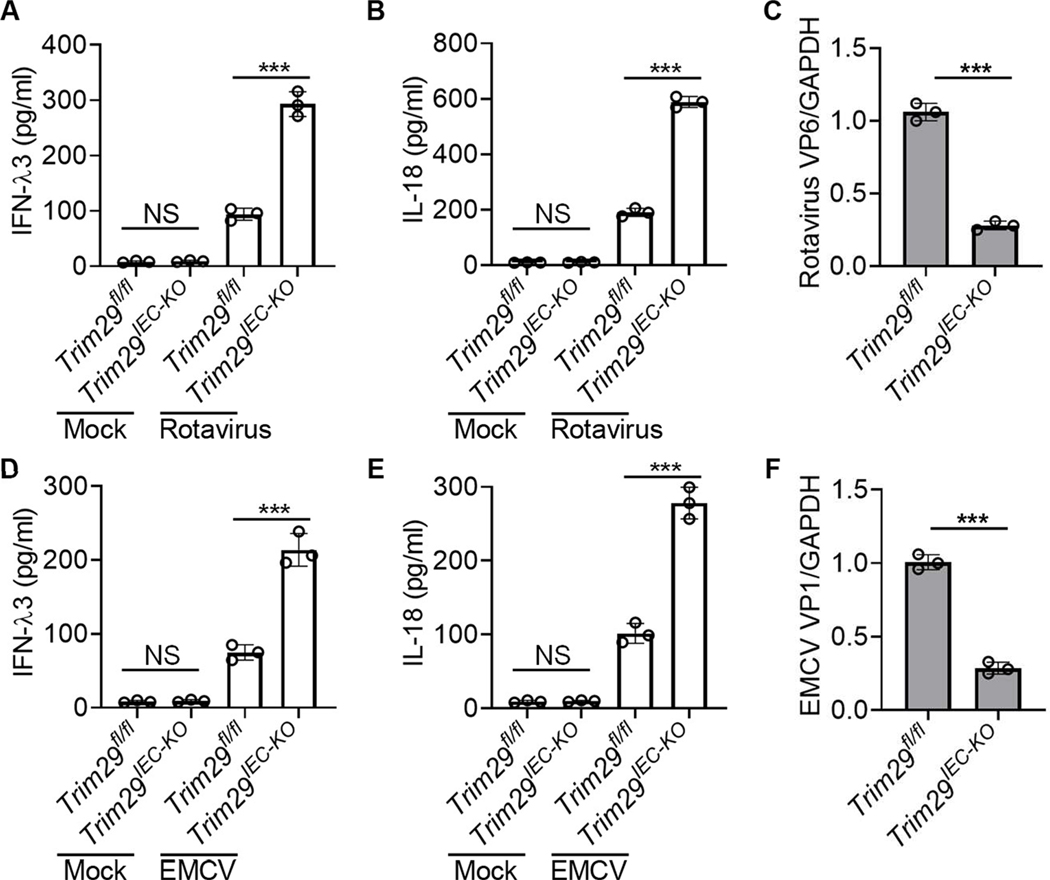
TRIM29 negatively regulates production of IFN-λ3 and IL-18 in mouse primary IECs upon enteric RNA virus infection. (**A-B,D-E**) ELISA of IFN-λ3 (**A, D**) and IL-18 (**B, E**) production in primary IECs from mouse intestinal organoids of wild-type *Trim29*^fl/fl^ and *Trim29*^*IEC-KO*^ mice after a 20 h infection without (Mock) or with enteric RNA viruses including simian rotavirus SA-11 strain (**A-B**) and EMCV K strain (**D-E**) at a MOI of 5. Mock, cells without virus infection. (**C,F**) Quantification of expression of rotavirus VP6 gene (**C**) and EMCV VP1 gene (**F**) relative to GAPDH in primary IECs from mouse intestinal organoids of wild-type *Trim29*^fl/fl^ and *Trim29*^*IEC-KO*^ mice infected by rotavirus (**C**) or EMCV (**F**) as in **A,D**. Data are represented as mean ± SD. NS (not significant), *P* > 0.05; ***P < 0.001 (unpaired *t* test). Data are representative of three technical replicates.

To further confirm the role of IEC-specific TRIM29 in mouse IECs during enteric RNA virus infections, we generated IEC-specific TRIM29 knockout mice. Initially, the *Trim29*-targetd mice were crossed with FRT deleter (Rosa26-FLPe) mice, resulting in *Trim29*-flox mice (*Trim29*^fl/fl^). These were subsequently mated with *Villin*-Cre transgenic mice to generate IEC-specific *Trim29*-knockout mice, *Trim29*^fl/fl^; *Villin*-Cre (*Trim29*^*IEC-KO*^) (Supplementary Fig. 2A). Deletion of *Trim29* was confirmed by PCR analysis of genomic DNA (Supplementary Fig. 2B). Moreover, mouse primary IECs were isolated from wild-type *Trim29*^fl/fl^ and *Trim29*^*IEC-KO*^ mice and immunoblot analysis confirmed that TRIM29 was deleted in mouse IECs from *Trim29*^*IEC-KO*^ mice (Supplementary Fig. 2C). In addition, flow cytometry analysis showed that the purity of primary IECs isolated from wild-type *Trim29*^fl/fl^ and *Trim29*^*IEC-KO*^ mice was more than 95 % (Supplementary Fig. 3) and knockout of TRIM29 in IECs did not change the expression of differentiation markers including EpCAM and E-Cadherin (Supplementary Fig. 3), indicating that TRIM29 does not affect expression of differentiation marker in mouse IECs. Additionally, IEC-specific TRIM29 deficiency did not affect the body weights of *Trim29*^fl/fl^ and *Trim29*^*IEC-KO*^ female and male mice (Supplementary Fig. 4A). Epithelial barrier function of IECs plays a crucial role in regulating the intestinal homeostasis and inflammation^32^. However, the mouse IECs from wild-type *Trim29*^fl/fl^ and *Trim29*^*IEC-KO*^ mice had a comparable level of expression of several major tight junction proteins, including E-cadherin, Claudin-2, Occludin, and zonula occludens-1(ZO-1) (Supplementary Fig. 4B-4E), suggesting that TRIM29 is dispensable for expression of epithelial tight junction proteins. These data show that the IEC-specific *TRIM29*-knockout mice are successfully generated for following *in vitro* and *in vivo* studies. Next, primary IECs of mouse intestinal organoids were cultured from wild-type *Trim29*^fl/fl^ and *Trim29*^*IEC-KO*^ mice and were infected without or with two enteric RNA viruses including simian rotavirus SA-11 strain and EMCV for detecting cytokine production by ELISA. Primary IECs from mouse intestinal organoids of *Trim29*^*IEC-KO*^ mice produced much more levels of IFN-λ3 and IL-18 than those from wild-type *Trim29*^fl/fl^ mice in response to enteric RNA virus rotavirus (Fig. 2A and 2B) or EMCV (Fig. 2D and 2E). However, both IFN-λ3, and IL-18 were barely produced in primary IECs from mouse intestinal organoids of both wild-type *Trim29*^fl/fl^ and *Trim29*^*IEC-KO*^ mice without infection (Fig. 2A–2B and 2D-2E). Furthermore, TRIM29 deficiency in primary IECs from mouse intestinal organoids restricted the viral replication of enteric RNA virus rotavirus (Fig. 2C) or EMCV (Fig. 2F). Taken together, our findings underscore that while TRIM29 is dispensable for barrier integrity, its deficiency in IECs restricts infections by enteric RNA viruses by enhancing IFN-λ3 and IL-18 production in primary IECs from mouse intestinal organoids.

### TRIM29 deficiency controls intestinal inflammation induced by enteric rotavirus infection in vivo

Rotavirus is the primary pathogen responsible for viral gastroenteritis^4^ and diarrheal mortality, causing > 200,000 deaths and millions of hospitalizations each year^3,33^. Our studies have revealed that TRIM29 deficiency restricts enteric RNA viruses by amplifying IFN-λ3, and IL-18 in both human and mouse IECs *invitro.* To investigate TRIM29′s role in modulating intestinal inflammation in a live setting, we employed a rotavirus-induced gastroenteritis model in suckling mice. Following oral rotavirus exposure, a large proportion of *Trim29*^+/+^ suckling mice displayed frequent diarrhoeal episodes. In contrast, the *Trim29*^−/−^ suckling mice showed markedly fewer diarrhoeal occurrences (Supplementary Fig. 5A) We next infected both wild-type *Trim29*^+/+^ and *Trim29*^−/−^ suckling mice with rotavirus orally for one day and subsequently quantified IFN-λ3 and IL-18 levels in intestine homogenates from infected mice. The *Trim29*^−/−^ suckling mice exhibited over 3-fold increase in IFN-λ3 and IL-18 production compared to their wild-type *Trim29*^+/+^ counterparts following rotavirus infection (Supplementary Fig. 5B and 5C). On day 5 post-infection, we extracted intestine samples and measured the viral titers of rotavirus using qRT-PCR. We detected higher viral loads in the intestine in wild-type *Trim29*^+/+^ suckling mice than in *Trim29*^−/−^ littermates (Supplementary Fig. 5D). These data indicate that the absence of TRIM29 offers suckling mice a protective shield against rotavirus-triggered intestinal inflammation *in vivo*.

**Fig. 3.**
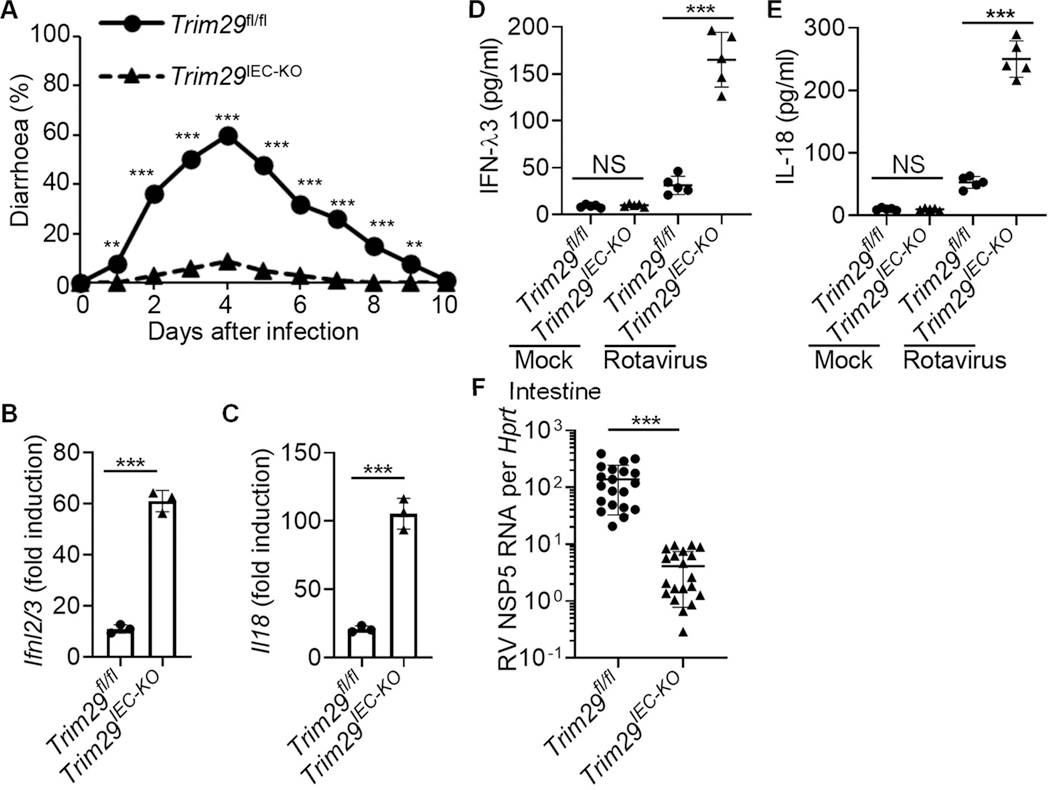
IEC-specific TRIM29 deficiency controls intestinal inflammation induced by enteric rotavirus infection in suckling mice *in vivo*. (**A**) Diarrhoea duration and percentage of mice with diarrhea (score ≥ 2) from 8-day-old wild-type *Trim29*^fl/fl^ and *Trim29*^*IEC-KO*^ suckling mice (*n* = 20 per strain) orally inoculated by gavage with 1 DD50 rotavirus EW strain. (**B-E**) The wild-type *Trim29*^fl/fl^ and *Trim29*^*IEC-KO*^ suckling mice (*n* = 3 or 5 per strain) were orally inoculated by gavage without (Mock) or with 1 DD50 rotavirus EW strain. At day 1 post-inoculation, mice were euthanized, and intestine tissues were excised for qRT-PCR detection of *Ifnl2/3* (**B**) and *Il18* (**C**) expression. Additionally, the excised intestine was homogenized in PBS for detection of IFN-λ3 (**D**) and IL-18 (**E**) in intestine homogenates by ELISA. (**F**) The wild-type *Trim29*^fl/fl^ and *Trim29*^*IEC-KO*^ suckling mice (*n* = 20 per strain) were orally inoculated by gavage with 1 DD50 rotavirus EW strain. On day 5 post-inoculation, mice were euthanized, and intestine tissues were collected for qRT-PCR detection of rotavirus levels. Mock, mouse without rotavirus infection. Data are represented as mean ± SD. NS, *P* > 0.05, **P < 0.01 and ***P < 0.001 (unpaired *t* test). Data are representative of three experiments.

Considering TRIM29′s pivotal role in regulating IFN-λ3 and IL-18 production in IECs, we proceeded to assess the significance of IEC-specific TRIM29 deficiency in controlling intestinal inflammation induced by enteric rotavirus infection using the *Trim29*^*IEC-KO*^ suckling mice *in vivo*. We first challenged wild-type *Trim29*^fl/fl^ and *Trim29*^*IEC-KO*^ suckling mice orally with mouse rotavirus and monitored diarrhoea over time. *Trim29*^*IEC-KO*^ suckling mice exhibited much less frequent incidences of diarrhoea compared to their wild-type *Trim29*^fl/fl^ littermates (Fig. 3A). In addition, we infected wild-type *Trim29*^fl/fl^ and *Trim29*^*IEC-KO*^ suckling mice orally with rotavirus for one day and then measured IFN-λ3 and IL-18 in intestine homogenates from infected mice. The qRT-PCR analysis showed that the expression of *Ifnl2/3 and Il18* in intestines of *Trim29*^*IEC-KO*^ suckling mice was significantly higher than that in intestines from wild-type *Trim29*^fl/fl^ littermates (Fig. 3Band 3C). Additionally, *Trim29*^*IEC-KO*^ suckling mice produced over 3-fold more IFN-λ3 and IL-18 than did wild-type *Trim29*^fl/fl^ littermates, in intestines with rotavirus infection (Fig. 3D and 3E). However, IFN-λ3 and IL-18 were barely expressed in intestines from both wild-type *Trim29*^fl/fl^ and *Trim29*^*IEC-KO*^ suckling mice without rotavirus infection (Fig. 3D and 3E). Furthermore, we harvested intestine samples on day 5 post-infection, and determined viral titers of rotavirus by qRT-PCR. We detected higher viral loads in the intestine in wild-type *Trim29*^fl/fl^ suckling mice than in *Trim29*^*IEC-KO*^ littermates (Fig. 3F). Together, these data demonstrate that IEC-specific TRIM29 deficiency is sufficient to control intestinal inflammation and infection by enteric rotavirus in suckling mice *in vivo*.

### TRIM29 ablation restricts intestinal inflammation induced by enteric EMCV infection in vivo

**Fig. 4.**
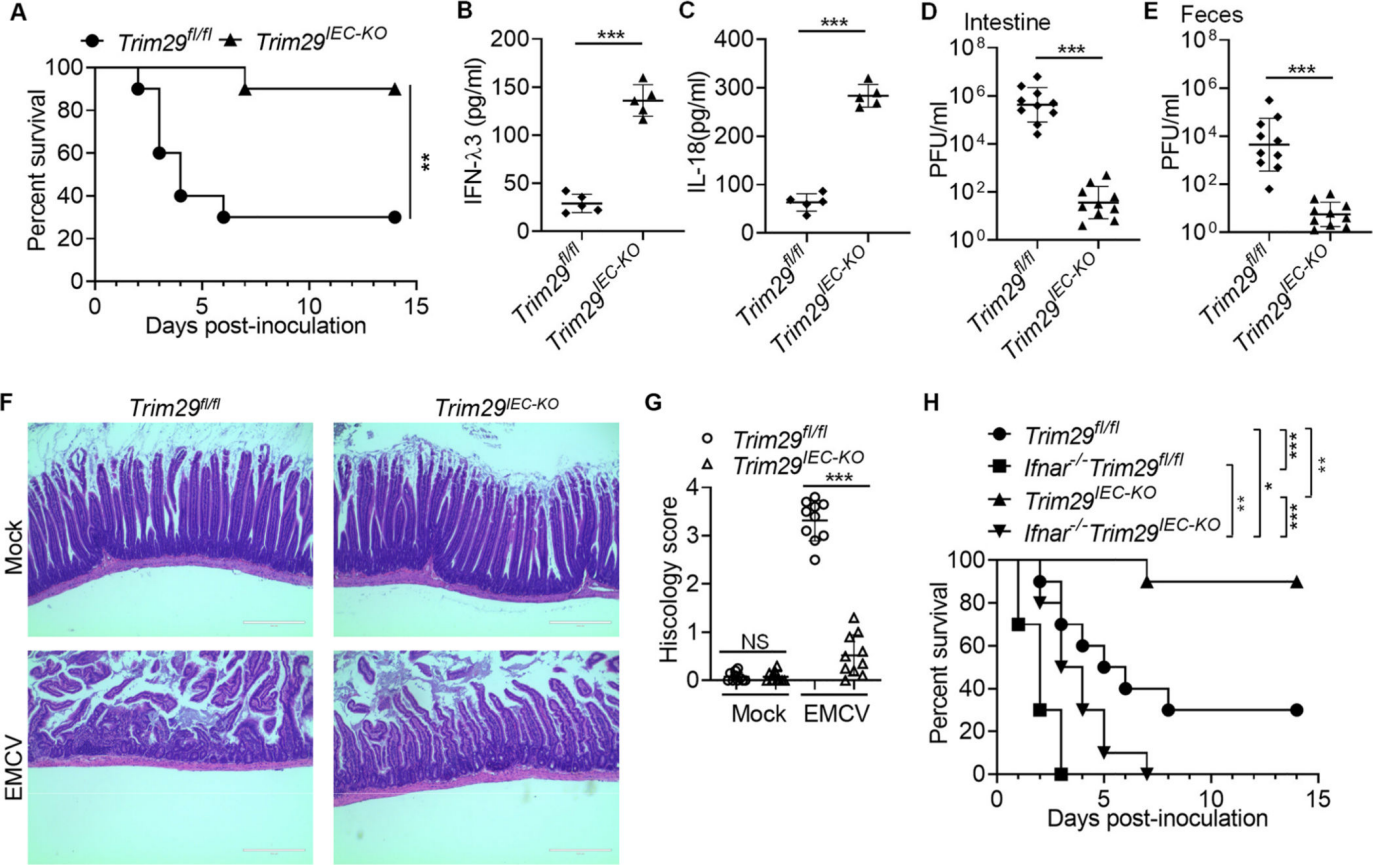
IEC-specific TRIM29 ablation restricts intestinal inflammation induced by enteric EMCV infection in adult mice *in vivo.* (**A**) Survival of five-week-old wild-type *Trim29*^fl/fl^ and *Trim29*^*IEC-KO*^ adult mice (*n* = 10 per strain) after intragastrical injection of EMCV (1 × 10^8^ plaque-forming units (PFU) per mouse). (**B,C**) The wild-type *Trim29*^fl/fl^ and *Trim29*^*IEC-KO*^ mice (*n* = 5 per strain) were inoculated intragastrically with 1 × 10^8^ PFU of EMCV. On day 1 post-inoculation, mice were euthanized, and intestine tissues were excised, and homogenized in PBS. Levels of IFN-λ3 (**B**) and IL-18 (**C**) in intestine homogenates was quantified by ELISA. (**D,E**) The wild-type *Trim29*^fl/fl^ and *Trim29*^*IEC-KO*^ mice (*n* = 10 per strain) were inoculated intragastrically with 1 × 10^8^ PFU of EMCV. On day 4 post-inoculation, mice were euthanized, feces were collected and intestine tissues were excised. The viral titers in intestine homogenates (**D**) and shedding in feces (**E**) were determined by plaque assay. Results are expressed as mean viral titers for 10 animals for each time point. Error bars indicate standard errors of the mean. (**F**) Hematoxylin and eosin (H&E)-staining of duodenum sections from wild-type *Trim29*^fl/fl^ and *Trim29*^*IEC-KO*^ mice as in (**D)**. Scale bars represent 200 μm. (**G**) Graph depicting histology scores for inflammation and tissue damage of duodenum sections with 10 different areas in (**F)**. (**H**) Survival of five-week-old wild-type *Trim29*^fl/fl^, *Trim29*^*IEC-KO*^, *Ifnar1*^*−/−-*^*Trim29*^*fl/fl*^ and *Ifnar1*^*−/−*^ *Trim29*^*IEC-KO*^ adult mice (*n* = 10 per strain) after intragastrical injection of EMCV (1 × 10^8^ plaque-forming units (PFU) per mouse). Data are represented as mean ± SD. NS, *P* > 0.05, **P < 0.01 and ***P < 0.001 (unpaired *t* test and Gehan-Breslow-Wilcoxon test for survival analysis). Data are representative of three experiments.

We next evaluated if TRIM29 regulates intestinal inflammation induced by enteric EMCV in adult mice *in vivo*. The five-week-old wild-type *Trim29*^fl/fl^ and *Trim29*^*IEC-KO*^ adult mice were inoculated intragastrically with enteric EMCV and mice survival was monitored over time. The challenge of wild-type *Trim29*^fl/fl^ mice with EMCV led to lethal infection (Fig. 4A). In contrast, most of *Trim29*^*IEC-KO*^ mice survived from EMCV infection (Fig. 4A). In addition, we infected wild-type *Trim29*^fl/fl^ and *Trim29*^*IEC-KO*^ mice intragastrically with enteric EMCV for one day and then measured IFN-λ3 and IL-18 in intestine homogenates from infected mice. As expected, *Trim29*^*IEC-KO*^ mice produced over 3-fold more IFN-λ3 and IL-18 than did wild-type *Trim29*^fl/fl^ mice, in response to EMCV (Fig. 4B and 4C). Additionally, we harvested intestine tissues and feces on day 4 post-infection, and determined viral titers of EMCV in those organs by plaque assay. We detected significantly higher viral loads in the intestine and more increased fecal shedding of EMCV in wild-type *Trim29*^fl/fl^ mice than in *Trim29*^*IEC-KO*^ mice (Fig. 4D and 4E). Furthermore, duodenum histopathology and histology score showed that the duodenum from wild-type *Trim29*^fl/fl^ mice exhibited more severe inflammatory lesions and inflammation compared to *Trim29*^*IEC-KO*^ mice on day 2 after EMCV infection (Fig. 4F and 4G). Previously, we have showed that TRIM29 plays critical roles in host defense against systemic infection of DNA and RNA viruses by negatively regulating type I IFN production through degrading innate immune adaptors such as STING^29^, NEMO^28^ and MAVS^34^. To exclude the possible role of TRIM29-regulated type I IFN in the antiviral phenotype during intestinal infection of enteric viruses, we used Ifnar1 (type I IFN receptor) knockout (*Ifnar1*^*−/−*^) mice and crossed them with *Trim29*^*fl/fl*^ and *Trim29*^*IEC-KO*^ mice to generate *Ifnar1*^*−/−*^*Trim29*^*fl/fl*^ and *Ifnar1*^*−/−*^ *Trim29*^*IEC-KO*^ mice. Next, *Trim29*^*fl/fl*^, *Trim29*^*IEC-KO*^, *Ifnar1*^*−/−*^*Trim29*^*fl/fl*^ and *Ifnar1*^*−/−*^ *Trim29*^*IEC-KO*^ mice were inoculated intragastrically with EMCV and those mice were monitored daily for survival. We found that compared with *Trim29*^*fl/fl*^ and *Trim29*^*IEC-KO*^ mice, the survival of *Ifnar1*^*−/−*^*Trim29*^*fl/fl*^ and *Ifnar1*^*−/−*^*Trim29*^*IEC-KO*^ mice were much worse (Fig. 4H), suggesting that type I IFN signal plays important role in controlling mice survival against enteric viruses, which is consistent with our recent report about critical role of RNA sensor DHX15-mediated type I IFN in host defense against enteric RNA viruses^18^. Importantly, compared with *Ifnar1*^*−/−–*^ *Trim29*^*fl/fl*^ mice, *Ifnar1*^*−/−*^ *Trim29*^*IEC-KO*^ mice survived much better from EMCV intestinal infection (Fig. 4H), suggesting that the antiviral phenotype of TRIM29 knockout mice results from TRIM29-mediated regulation of type III IFN and inflammasome activation. These collective findings indicate that IEC-specific TRIM29 ablation restricts intestinal inflammation induced by enteric EMCV infection in adult mice *in vivo*.

### TRIM29 knockout recruits more intraepithelial Ly6A^+^ CCR9^+^ CD4^+^ T cells to protect against enteric viral infection

**Fig. 5.**
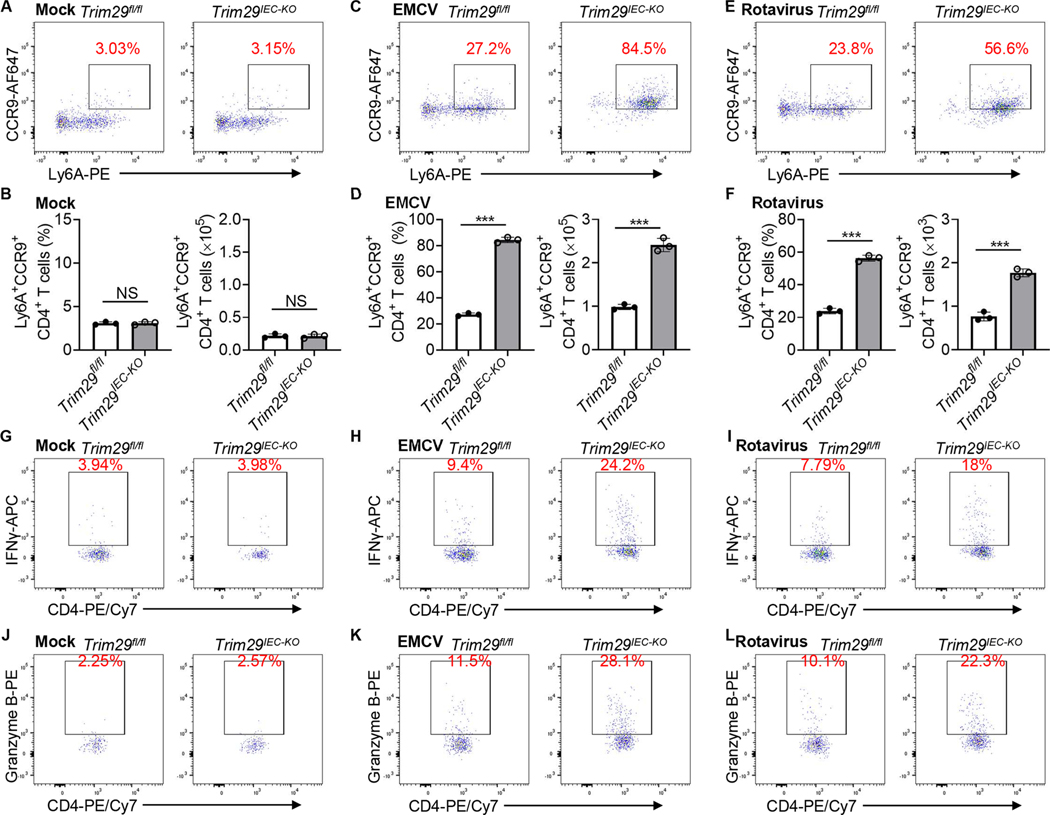
TRIM29 knockout recruits more intraepithelial Ly6A^+^CCR9^+^CD4^+^ T cells to protect against enteric viral infection. (**A-F**) Flow cytometry (**A, C, E**), percent and absolute cell number quantification (**B, D, F**) analysis of mouse intraepithelial Ly6A^+^CCR9^+^CD4^+^ T in small intestine intraepithelial lymphocytes from both *Trim29*^fl/fl^ and *Trim29*^*IEC-KO*^ adult mice infected without (Mock, **A, B**) or with EMCV (**C, D**), or both *Trim29*^fl/fl^ and *Trim29*^*IEC-KO*^ suckling mice infected with Rotavirus (**E, F**) for 3 days using Ly6A-PE and CCR9-AF647 antibodies. (**G-L**) Flow cytometry analysis of mouse intraepithelial IFN-γ producing CD4^+^ T cells (IFN-γ^+^ CD4^+^ T cells, **G-I**) or Granzyme B producing CD4^+^ T cells (Granzyme B^+^ CD4^+^ T cells, **J-L**) in small intestine intraepithelial lymphocytes from both *Trim29*^fl/fl^ and *Trim29*^*IEC-KO*^ adult mice infected without (Mock, **G, J**) or with EMCV (**H, K**), or both *Trim29*^fl/fl^ and *Trim29*^*IEC-KO*^ suckling mice infected with Rotavirus (**I, L**) for 3 days using CD4-PE/Cy7 and IFN-γ-APC or Granzyme B-PE antibodies. Flow cytometry data were acquired on an LSR-II flow cytometer (Beckton Dickinson) and analyzed using FlowJo v10 software (Tree Star). Data are shown as the mean ± SD. NS, *P* > 0.05, and ***P < 0.001 (unpaired *t* test). Data are representative of three experiments.

Intraepithelial lymphocytes (IELs) are a prominent T cell population situated at the pivotal juncture between the intestinal lumen and the core of the body^35,36^. IELs serve as a first line of immunity in mice and humans, striking a balance between tolerance and defense^37,38,39,40^. It’s reported that there are newly recruited intraepithelial Ly6A^+^CCR9^+^CD4^+^ T cells in small intestines after infection with enteric viruses including murine norovirus and adenovirus, and those intraepithelial Ly6A^+^CCR9^+^CD4^+^ T cells play protective role in host defense against enteric viral infection by producing IL-18-dependent protective IFN-γ^27^. Given the critical role of TRIM29 in IL-18 production in IECs both *in vitro* and *in vivo* during infection of enteric RNA viruses including EMCV and rotavirus, we next investigated whether the increased IL-18 in IECs from *Trim29*^IEC-KO^ mice augments the recruitment of intraepithelial Ly6A^+^CCR9^+^CD4^+^ T cells in small intestines during enteric RNA virus infection *in vivo*. Flow cytometry analysis showed comparable frequencies and cell numbers of intraepithelial Ly6A^+^CCR9^+^CD4^+^ T cells in small intestines from *Trim29*^fl/fl^ and *Trim29*^IEC-KO^ adult mice (Fig. 5A and 5B) before EMCV infection or from *Trim29*^fl/fl^ and *Trim29*^IEC-KO^ suckling mice (Supplementary Fig. 6A) before rotavirus infection. However, higher frequencies and cell numbers of intraepithelial Ly6A^+^CCR9^+^CD4^+^ T cells were infiltrated in small intestines from *Trim29*^IEC-KO^ adult mice than those from *Trim29*^fl/fl^ mice after EMCV infection for 3 days (Fig. 5C and 5D). Similarly, there were more frequencies and cell numbers of intraepithelial Ly6A^+^CCR9^+^CD4^+^ T cells in small intestines from *Trim29*^IEC-KO^ suckling mice than those from *Trim29*^fl/fl^ mice after rotavirus infection for 3 days (Fig. 5E And 5F). Because TRIM29 controls IFN-λ3 and IL-18 production in IECs, we next investigated if cytokines IFN-λ3 and IL-18 are required for recruitment of intraepithelial Ly6A^+^CCR9^+^CD4^+^ T cells in small intestines during enteric virus infection. We found that administration of IL-18 binding protein (IL-18BP), but not IFNLR1 blocking peptide (IFNLR1 BP), impaired the recruitment of intraepithelial Ly6A^+^CCR9^+^CD4^+^ T cells in small intestines with PBS control in both *Trim29*^*fl/fl*^ and *Trim29*^*IEC-KO*^ adult mice infected with EMCV (Supplementary Fig. 6B), suggesting that IL-18 is required for recruitment of intraepithelial Ly6A^+^CCR9^+^CD4^+^ T cells in small intestines during enteric virus infection. The newly recruited intraepithelial CD4^+^ T cells are shown to play protective role in host defense against enteric adenovirus infection by producing protective IFN-γ and Granzyme B^27^. Thus, we investigated whether intraepithelial CD4^+^ T cells in small intestines from *Trim29*^IEC-KO^ mice elicited antiviral functions by assessing the production of protective IFN-γ and Granzyme B by intraepithelial CD4^+^ T cells. Flow cytometry analysis revealed similar frequencies of intraepithelial IFN-γ-expressing CD4^+^ T cells (Fig. 5G and Supplementary Fig. 6C) and Granzyme B-expressing CD4^+^ T cells (Fig. 5J and Supplementary Fig. 6F) in small intestines from *Trim29*^fl/fl^ and *Trim29*^IEC-KO^ mice before EMCV infection (Supplementary Fig. 6G). However, *Trim29*^IEC-KO^ adult mice had significantly higher frequencies of intraepithelial IFN-γ-expressing CD4^+^ T cells (Fig. 5H and Supplementary Fig. 6D) and Granzyme B-expressing CD4^+^ T cells (Fig. 5K and Supplementary Fig. 6G) in small intestines than *Trim29*^fl/fl^ mice after EMCV infection for 3 days. Similarly, there were more frequencies of intraepithelial IFN-γ-expressing CD4^+^ T cells (Fig. 5I and Supplementary Fig. 6E) and Granzyme B-expressing CD4^+^ T cells (Fig. 5L and Supplementary Fig. 6H) in small intestines from *Trim29*^IEC-KO^ suckling mice than those from *Trim29*^fl/fl^ mice after rotavirus infection for 3 days. Taken together, these data demonstrate that TRIM29 deficiency in IECs recruits more intraepithelial Ly6A^+^CCR9^+^CD4^+^ T cells in small intestines to protect against enteric RNA virus infection through elevated production of protective IFN-γ and Granzyme B *in vivo*.

### TRIM29 interacts with NLRP6 and NLRP9b to inhibit IFN-λ3 and IL-18 production

**Fig. 6.**
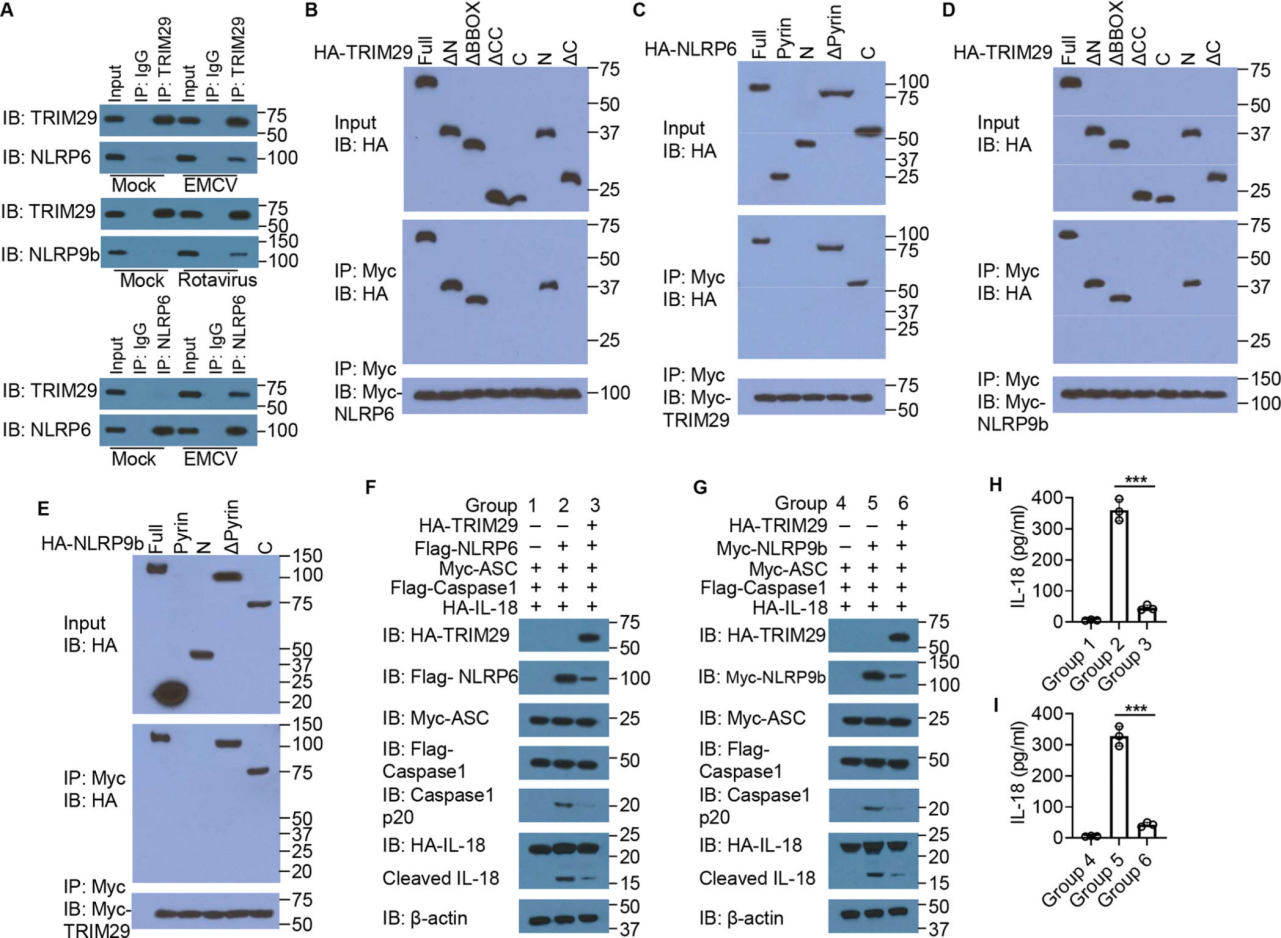
TRIM29 interacts with NLRP6 and NLRP9b to suppress inflammasome activation. (**A**) Immunoblot analysis of endogenous proteins of TRIM29 and NLRP6 or NLRP9b precipitated with anti-TRIM29 or anti-NLRP6 from whole-cell lysates of in primary IECs from mouse intestinal organoids of wild-type *Trim29*^fl/fl^ mice infected without (Mock) or with Rotavirus and EMCV at MOI of 5 with proteasome inhibitor MG132 (5 μM) treatment before lysis. (**B,D**) Immunoblot analysis (with anti-HA) of purified HA-tagged full-length TRIM29 (Full) and TRIM29 truncation mutants alone (*top blot*) or after incubation with Myc-tagged NLRP6 (**B**) or NLRP9b (**D**) and immunoprecipitation with anti-Myc antibody (*middle blot*), and immunoblot analysis (with anti-Myc) of Myc-tagged NLRP6 (**B**) or NLRP9b (**D**) after incubation with Myc-tagged NLRP6 (**B**) or NLRP9b (**D**) with anti-Myc antibody (*bottom blot*). (**C**) Immunoblot analysis (with anti-HA) of purified HA-tagged full-length NLRP6 (Full) and NLRP6 truncation mutants alone (*top blot*) or after incubation with Myc-tagged TRIM29 and immunoprecipitation with anti-Myc antibody (*middle blot*), and immunoblot analysis (with anti-Myc) of Myc-tagged TRIM29 after incubation with Myc-tagged TRIM29 with anti-Myc antibody (*bottom blot*). (**E**) Immunoblot analysis (with anti-HA) of purified HA-tagged full-length NLRP9b (Full) and NLRP9b truncation mutants alone (*top blot*) or after incubation with Myc-tagged TRIM29 and immunoprecipitation with anti-Myc antibody (*middle blot*), and immunoblot analysis (with anti-Myc) of Myc-tagged TRIM29 after incubation with Myc-tagged TRIM29 with anti-Myc antibody (*bottom blot*). (**F,G**) Immunoblot analysis of TRIM29, NLRP6 (**F**) or NLRP9b (**G**), ASC, caspase-1, cleaved caspase1 p20, full-length IL-18, and its cleaved IL-18 in HEK293T cells transfected with HA-IL-18, Myc-ASC, and FLAG-caspase-1, and co-transfected with or without Flag-NLRP6 (**F**) or Myc-NLRP9b (**G**), and HA-TRIM29. The position of protein markers (shown in kDa) is indicated at right. (**H,I**) ELISA of IL-18 production in supernatant of HEK293T cells of groups 1–3 (**H**) and groups 4–6 (**I**). Data are representative of three technical replicates.

We and others have previously shown that RNA helicase DHX15 senses double strand RNA of enteric RNA viruses to recruit downstream adaptors NLRP6 for both MAVS signaling and inflammasome activation to induce production of IFN-λ3 and IL-18 in dendritic cells and IECs^18,17,36^. Additionally, the NLRP9b inflammasome is shown to restrict rotavirus infection in IECs *in vivo* by recruiting another RNA helicase DHX9 to detect dsRNA of rotavirus for producing antiviral IL-18^26^. Next, we determined the molecular mechanisms by which TRIM29 inhibits inflammasome activation and cytokine production in IECs following enteric RNA virus infection. We first determined the possible interactions of TRIM29 with NLRP6 and NLRP9b in IECs at the endogenous protein level, because only NLRP6 and NLRP9b, but not other members of the NLR family like NLRP3, have been identified as being selectively expressed in mouse IECs^26^. Anti-TRIM29 Ab, but not control IgG, could pull down NLRP6 and NLRP9b in primary IECs from mouse intestine organoids of *Trim29*^fl/fl^ mice infected by EMCV and Rotavirus, respectively. Similarly, anti-NLRP6 Ab, but not control IgG, could pull down TRIM29 in mouse primary IECs infected by EMCV. By contrast, anti-TRIM29 Ab could not pull down NLRP6 or NLRP9b without infection, suggesting there were indeed interactions of TRIM29 with NLRP6 and NLRP9b in primary IECs from mouse intestine organoids under the condition of enteric RNA virus infection (Fig. 6A). Additionally, although NLRP6 and NLRP9b were barely expressed in human HT-29 IECs, NLRP6 and NLRP9b were induced in HT-29 IECs after EMCV and rotavirus infection (Supplementary Fig. 7A), respectively, suggesting thatNLRP6 and NLRP9b control the upregulation of IFN-λ3 and IL-18 upon knock down of TRIM29. To further map the binding sites between TRIM29 and NLRP6, we analyzed the interactions between Myc-tagged recombinant NLRP6 and HA-tagged recombinant full-length TRIM29 and truncation mutants of TRIM29 (Supplementary Fig. 7B). Both full-length TRIM29 and the CC (coiled coil) and OmpH (outer membrane protein H) domains of TRIM29 bound NLRP6 (Fig. 6B). Additionally, the mapping results for Myc-tagged recombinant TRIM29 and HA-tagged full-length NLRP6 and their truncation mutants (Supplementary Fig. 7C) showed that the PPP1R42 (Protein Phosphatase 1 Regulatory Subunit 42) domain of NLRP6 bound TRIM29 (Fig. 6C). To further identify the binding sites between TRIM29 and NLRP9b, we investigated the interactions between Myc-tagged recombinant NLRP9b and HA-tagged recombinant full-length TRIM29 and truncation mutants of TRIM29 (Supplementary Fig. 7B). Both full-length TRIM29 and the CC (coiled coil) and OmpH (outer membrane protein H) domains of TRIM29 bound NLRP9b (Fig. 6D). Additionally, the mapping results for Myc-tagged recombinant TRIM29 and HA-tagged full-length NLRP9b and their truncation mutants (Supplementary Fig. 7D) showed that the Protein Phosphatase 1 Regulatory Subunit 42 (PPP1R42) domain of NLRP9b bound TRIM29 (Fig. 6E). These results indicate that TRIM29 uses its CC and OmpH domains to interact with the PPP1R42 domain of NLRP6 and NLRP9b, respectively. Inflammasome assembly and activation are essential for IL-18 maturation^41,42^. To further investigate whether TRIM29 interacts with NLRP6 or NLRP9b to regulate NLRP6 or NLRP9b inflammasome activation for IL-18 maturation, we reconstituted NLRP6 (Fig. 6F) or NLRP9b (Fig. 6G) inflammasome components in HEK293T cells. In the reconstituted system, NLRP6 (Fig. 6F) or NLRP9b (Fig. 6G) inflammasome activation resulted in caspase 1 cleavage, IL-18 cleavage and maturation, and IL-18 production (Fig. 6H and 6I). The overexpression of full-length TRIM29 significantly reduced NLRP6 (Fig. 6F) or NLRP9b (Fig. 6G) inflammasome activation by inducing much less caspase 1 cleavage, IL-18 cleavage (Fig. 6F and 6G), and IL-18 production (Fig. 6H and 6I), suggesting that TRIM29 interacts with NLRP6 and NLRP9b to inhibit caspase 1 cleavage, IL-18 maturation and production. Collectively, these data suggest that TRIM29 interacts with NLRP6 and NLRP9b to inhibit their inflammasome activation.

### TRIM29 ubiquitinates and degrades NLRP6 and NLRP9b

**Fig. 7.**
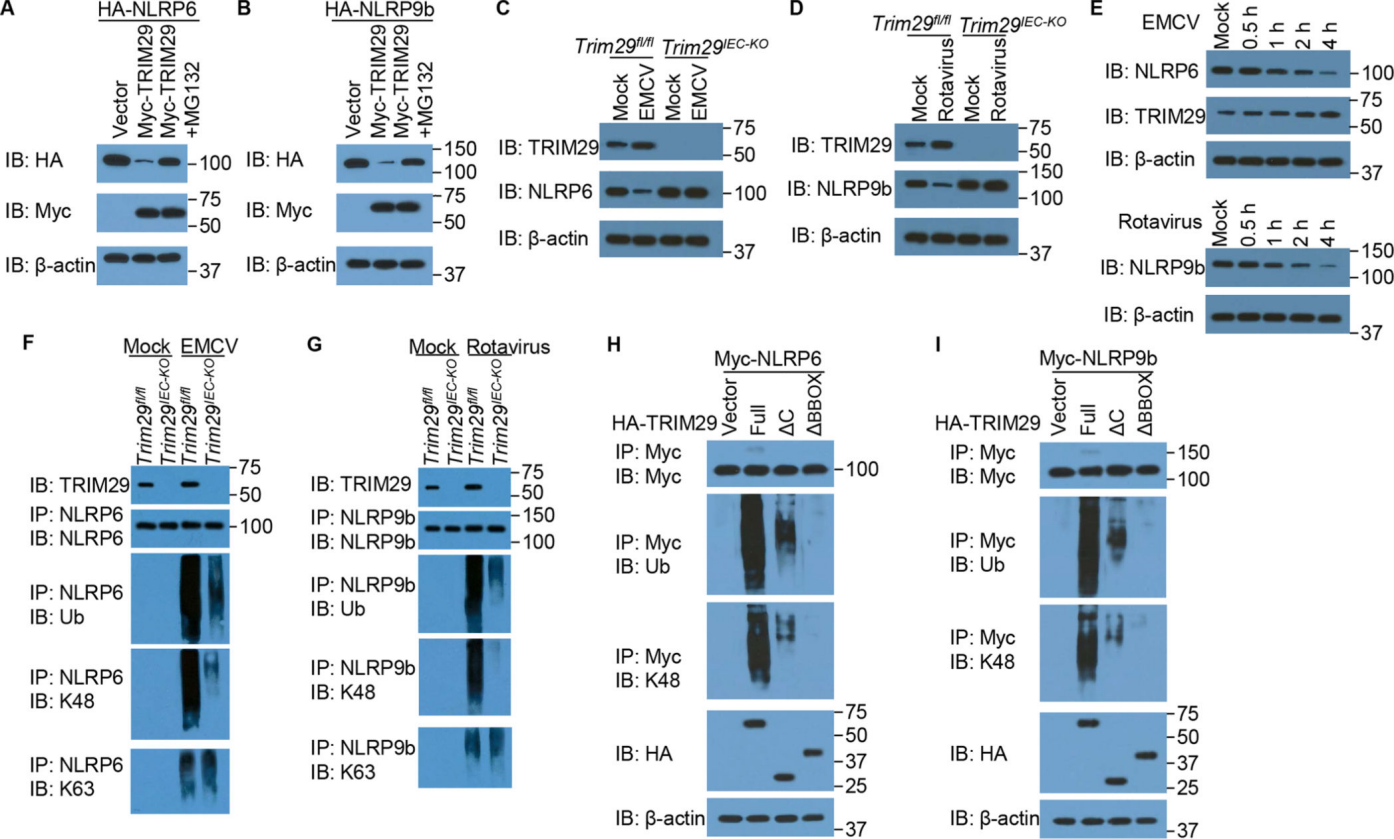
TRIM29 ubiquitinates and degrades NLRP6 and NLRP9b. (**A,B**) Immunoblot analysis of HA-tagged NLRP6 (**A**) or NLRP9b (**B**) (top blot), Myc-tagged TRIM29 (*middle blot*) and β-actin (*bottom blot*) in HEK293T cells co-transfected with expression vector for HA-tagged NLRP6 (**A**) or NLRP9b (**B**) and with empty vector or expression vector for Myc-tagged TRIM29, with or without treatment of 5 μM MG132 (above lanes). (**C,D**) Immunoblot analysis of TRIM29 (top blot), NLRP6 (**C**) or NLRP9b (**D**) (middle blot) and β-actin (bottom blot) in primary IECs from mouse intestinal organoids of wild-type *Trim29*^fl/fl^ and *Trim29*^*IEC-KO*^ mice infected with EMCV (**C**) or Rotavirus (RV, **D**) at MOI of 5 for 4 h. (**E**) Immunoblot analysis of NLRP6 and TRIM29 (top panel) and NLRP9b (bottom panel) in primary IECs from mouse intestinal organoids of wild-type *Trim29*^fl/fl^ mice infected without (Mock) or with EMCV (top panel) or Rotavirus (bottom panel) at MOI of 5 for 0.5 h, 1 h, 2 h or 4 h. (**F,G**) Immunoblot analysis of TRIM29 in primary IECs from mouse intestinal organoids of wild-type *Trim29*^fl/fl^ and *Trim29*^*IEC-KO*^ mice (top), and of the abundance (second blot), total ubiquitination (third blot), K48-mediated ubiquitination (fourth blot) and K63-mediated ubiquitination (bottom blot) of NLRP6 (**F**) or NLRP9b (**G**) in those cells, infected without (Mock) or with EMCV (**F**) or Rotavirus (**G**) at MOI of 5 for 4 h with proteasome inhibitor MG132 (5 μM) treatment before lysis, assessed after immunoprecipitation with anti-NLRP6 (**F**) or NLRP9b (**G**) antibody. (**H,I**) Immunoblot analysis (with anti-Myc) of the abundance (top), total ubiquitination (second blot), and K48-linked ubiquitination (third blot) of Myc-tagged NLRP6 (**H**) or NLRP9b (**I**) in HEK293T cells transfected with empty vector or expression vector for HA-tagged TRIM29, truncation T29-ΔC (losing binding site of NLRP6 or NLRP9b), truncation T29-ΔBBOX (losing E3 ubiquitin ligase activity of TRIM29), and infected with EMCV (**H**) or Rotavirus (**I**) at MOI of 5 for 4 h with proteasome inhibitor MG132 (5 μM) treatment before lysis, assessed after immunoprecipitation with anti-Myc; immunoblot analysis of whole-cell lysates with anti-HA (fourth blot) and anti-β-actin (bottom). The position of protein markers (shown in kDa) is indicated on the right.

Because TRIM29 is an E3 ubiquitin ligase^28,29,34,43^, we next investigated whether TRIM29 targets NLRP6 and NLRP9b for degradation through protein ubiquitination. We first co-expressed Myc-TRIM29 or vector control with HA-NLRP6 or HA-NLRP9b in HEK293T cells with or without treatment of MG132 and analyzed the expression of TRIM29, NLRP6 and NLRP9b. As a result, TRIM29 could significantly degrade the target protein NLRP6 (Fig. 7A) and NLRP9b (Fig. 7B), compared with the vector control. Additionally, the treatment of MG132 could rescue the expression of NLRP6 (Fig. 7A) and NLRP9b (Fig. 7B). To further determine whether the expressions of TRIM29, NLRP6 and NLRP9b were regulated during enteric RNA virus infection *in vitro*, we infected primary IECs from mouse intestine organoids of *Trim29*^fl/fl^ and *Trim29*^*IEC-KO*^ mice with EMCV or Rotavirus, respectively, and then measured the expressions of TRIM29, NLRP6 and NLRP9b in IECs after virus infection. TRIM29 was upregulated in primary IECs from mouse intestine organoids of *Trim29*^fl/fl^ mice after EMCV or Rotavirus infection (Fig. 7C and 7D), whereas NLRP6 (Fig. 7C) and NLRP9b (Fig. 7D) were downregulated in mouse IECs from *Trim29*^fl/fl^ mice after virus infection owing to the degradation by TRIM29. Meanwhile, NLRP6 (Fig. 7C) and NLRP9b (Fig. 7D) were upregulated in mouse IECs from *Trim29*^*IEC-KO*^ mice after virus infection owing to the loss of degradation by TRIM29. Next, we further investigated if TRIM29 mediates degradation of NLRP6 and NLRP9b in enteric virus infected intestines *in vivo*, we inoculated intragastrically *Trim29*^fl/fl^ and *Trim29*^*IEC-KO*^ mice with EMCV or Rotavirus, respectively, and then measured the expressions of TRIM29, NLRP6 and NLRP9b in IECs of small intestines after 2 days of virus infection. TRIM29 was upregulated in IECs from intestines of *Trim29*^fl/fl^ mice after EMCV or Rotavirus infection *in vivo* (Supplementary Fig. 8A), whereas NLRP6 and NLRP9b were downregulated in IECs of intestines from *Trim29*^fl/fl^ mice after virus infection owing to the degradation by TRIM29 (Supplementary Fig. 8A). On contrast, NLRP6 and NLRP9b were upregulated in IECs of intestines from *Trim29*^*IEC-KO*^ mice after virus infection owing to the loss of degradation by TRIM29 *in vivo* (Supplementary Fig. 8A). In addition, the reduction of NLRP6 and NLRP9b expression and upregulation of TRIM29 were first observed on 1 h in primary IECs from mouse intestine organoids of *Trim29*^fl/fl^ mice after infection of EMCV or Rotavirus (Fig. 7E). It is reported that NLRP6 is an IFN stimulated gene (ISG)^19^. We found that both NLRP6 and NLRP9b were induced in primary IECs from mouse intestine organoids of *Trim29*^fl/fl^ mice after infection of EMCV or Rotavirus (Supplementary Fig. 8B), further confirming that NLRP6 and NLRP9b are one of ISG and the degradation of NLRP6 and NLRP9b are mediated by TRIM29. Although TRIM29 had different expression patterns to NLRP6 and NLRP9b in other tissues, such as skin, TRIM29 showed a similar intestine expression pattern to NLRP6 and NLRP9b (Supplementary Fig. 8C). These data suggest that NLRP6 and NLRP9b are indeed the ubiquitination target of TRIM29 and TRIM29 targets NLRP6 and NLRP9b for degradation through ubiquitin–proteasome pathway in IECs both *in vitro* and *in vivo*.

To determine whether TRIM29 was responsible for the ubiquitination of NLRP6 and NLRP9b *ex vivo*, we infected primary IECs from mouse intestine organoids of *Trim29*^fl/fl^ and *Trim29*^*IEC-KO*^ mice without or with EMCV or Rotavirus for 6 h, respectively. Cell lysates were prepared and analyzed for the ubiquitination of NLRP6 and NLRP9b. In mouse IECs from *Trim29*^fl/fl^ mice, both NLRP6 (Fig. 7F) and NLRP9b (Fig.7G) underwent more pronounced K48-linked ubiquitination when compared with IECs from *Trim29*^*IEC-KO*^ mice following with EMCV or rotavirus infection. Although K63-linked ubiquitination of NLRP6 and NLRP9b was detected in IECs with EMCV or rotavirus infection, which aligns with previous report about K63-linked ubiquitination of NLRP6^44^, there was no difference of K63-linked ubiquitination in mouse IECs from *Trim29*^fl/fl^ and *Trim29*^*IEC-KO*^ mice (Fig. 7F and 7G). However, there was no obvious ubiquitination of NLRP6 and NLRP9b in mouse IECs from *Trim29*^fl/fl^ and *Trim29*^*IEC-KO*^ mice without infection (Fig. 7F and 7G). To investigate whether the ubiquitination of NLRP6 and NLRP9b were dependent on the binding site of TRIM29 with NLRP6 and NLRP9b, we transfected the HEK293T cells to co-express Myc-tagged NLRP6 (Fig. 7H) or NLRP9b (Fig. 7I) and HA-tagged full-length TRIM29, truncated TRIM29 lacking the binding site of NLRP6 and NLRP9b (T29-ΔC) or TRIM29 mutant lacking the E3 ubiquitin ligase activity (T29-ΔBBOX). We infected the cells for 6 h with EMCV (Fig. 7H) or Rotavirus (Fig. 7I), and then prepared cell lysates and incubated them for 5 min at 90 ^◦^C with 1 % SDS (sodium dodecyl sulfate) to disrupt protein–protein interactions, followed by immunoprecipitation of Myc-tagged NLRP6 or NLRP9b. Immunoblot analysis of HA or ubiquitin demonstrated that the ubiquitination of NLRP6 (Fig. 7H) or NLRP9b (Fig. 7I) was strongly enhanced by overexpression of TRIM29 but not by overexpression of T29-ΔC or T29-ΔBBOX. Immunoblot analysis of K48-linked ubiquitin further demonstrated that TRIM29 induced ubiquitination of NLRP6 (Fig. 7H) or NLRP9b (Fig. 7I) by K48-mediated linkage. Together, these data indicated that TRIM29 targeted NLRP6 and NLRP9b and induced their ubiquitination for protein degradation by K48-linkage.

## Discussion

**Fig. 8.**
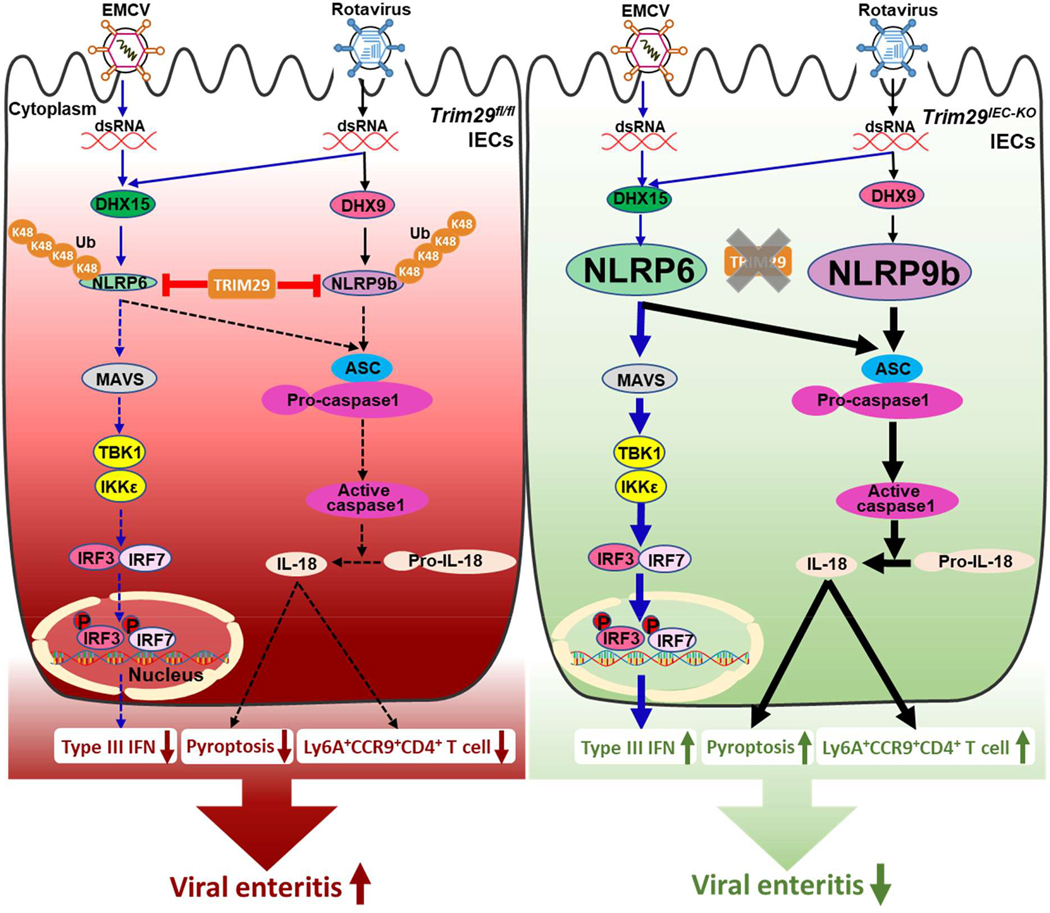
Working model for TRIM29-mediated controlling of enteric RNA virus-induced intestinal inflammation by targeting NLRP6 and NLRP9b signaling pathways. In *Trim29*^*fl/fl*^ IECs after infection with rotavirus and EMCV, TRIM29 interacts with NLRP6 and NLRP9b and targets them for K48-linked ubiquitination and degradation to reduce IFN-λ3 production and IL-18-mediated pyroptosis in IECs and decrease the recruitment of protective intraepithelial Ly6A^+^CCR9^+^CD4^+^ T cells in small intestines, which act in combination to cause viral enteritis. In contrast, in *Trim29*^*IEC-KO*^ IECs, without TRIM29-mediated degradation, NLRP6 and NLRP9b strongly promote IFN-λ3 production and IL-18-mediated pyroptosis in IECs and increase the recruitment of protective intraepithelial Ly6A^+^CCR9^+^CD4^+^ T cells in small intestines, which significantly reduce viral enteritis.

The host response to enteric viruses occurs primarily within the mucosa, where the IECs and intestinal immune system must balance protection against pathogens with tissue protection and tolerance to harmless commensal bacteria and food^3^. However, our understanding of the antiviral immunity processes within IECs, as well as the interplay between IECs and the intestinal immune system, remains in its infancy. In this study, we found that E3 ligase TRIM29 suppressed IFN-λ3 and IL-18 production, which in turn restricted the replication of enteric RNA viruses in human IECs *in vitro* when exposed to enteric RNA viruses including rotavirus and EMCV (Fig. 8). To further investigate the *in vivo* roles of TRIM29 in intestinal antiviral response and enteric virus induced intestinal inflammation, we generated the *Trim29* floxed mice (*Trim29*^fl/fl^) and IEC-specific *TRIM29*-knockout mice (*Trim29*^*IEC-KO*^). We found that while TRIM29 was dispensable for barrier integrity, its deficiency in IECs restricted enteric RNA viruses by enhancing IFN-λ3 and IL-18 production in mouse primary IECs. Importantly, IEC-specific *TRIM29*-knockout mice were resistant to infection with enteric rotavirus in suckling mice and EMCV in adult mice *in vivo*. The reduced intestinal inflammation induced by enteric viruses in IEC-specific *TRIM29*-deficient mice might be owing to enhanced production of IFN-λ3 and IL-18 in IECs and more recruitment of intraepithelial protective Ly6A^+^CCR9^+^CD4^+^ T cells in small intestines. Recently, NLRP6 has been shown to control enteric RNA virus, such as EMCV, induced intestinal inflammation through binding viral RNA via RNA sensor DHX15 and interacting with MAVS for producing antiviral IFN-λ and IL-18 in IECs^18,19^. In addition, the NLRP9b inflammasome is shown to restrict rotavirus infection in IECs *in vivo* by recruiting another RNA helicase DHX9 to detect dsRNA of rotavirus for producing antiviral IL-18^26^. Since NLRP6 and NLRP9b have been identified as being selectively expressed in mouse IECs^26^, we hypothesize that TRIM29 may interact with NLRP6 and NLRP9b to control IL-18 production in mouse IECs during enteric RNA virus infection. As expected, we demonstrated that TRIM29 interacted with NLRP6 and NLRP9b to target them for degradation through K48-linked ubiquitination, thereby preventing inflammasome assembly and activation, resulting in decreased IL-18 secretion in IECs. Therefore, we demonstrate that IEC specific TRIM29 deficiency is enough to control intestinal inflammation induced by enteric RNA viruses *in vivo*. Our findings indicate promise for potential therapeutic applications involving targeting E3 ligase TRIM29 to reduce intestinal inflammation in order to treat intestinal diseases such as viral gastroenteritis.

Type III IFN are largely produced by epithelial cells including IECs and type III IFN receptor is mainly expressed in epithelial cells^45^. Type III IFN and ISG expression in the intestine are required to control EMCV infection *in vivo*^19^, and IFN-λ system is essential for efficient control of rotavirus replication in IECs^46^. In present study, IEC-specific *TRIM29*-knockout mice are resistant to intestinal inflammation induced by either enteric rotavirus in suckling mice or EMCV in adult mice *in vivo*, which owes to the significant enhanced IFN-λ3 production by IECs from *TRIM29*-deficient mice. Our findings further confirm the important role of IFN-λ in controlling enteric virus infection in IECs and enteric virus-induced intestinal inflammation.

The inflammasome is a caspase-1 containing complex that activates the proinflammatory cytokines IL-1β and IL-18 and results in the proinflammatory cell death known as pyroptosis ^47,48^. Increasing evidence have highlighted the importance of inflammasome activation in the control of virus infection ^26,47^. We found that the inflammasome-derived cytokine IL-18 in intestine from IEC-specific *TRIM29*-deficient mice with enteric RNA virus infection was dramatically increased in either suckling mice or adult mice, indicating that inflammasome activation in IECs is important to control enteric RNA virus infection and virus induced intestinal inflammation. We found that TRIM29 suppresses IL-18 production in both human and mouse IECs in response to enteric RNA viruses including rotavirus and EMCV *in vitro* and *in vivo*. Mechanistically, we demonstrate that TRIM29 interacts with NLRP6 and NLRP9b to target them for degradation through K48-linked ubiquitination, thereby preventing inflammasome assembly and activation, resulting in decreased IL-18 secretion in IECs.

Components of the NLRP6 inflammasome are highly expressed in both large and small intestines and orchestrate the interface among host and microbes in the large intestine through controlling mucus secretion from goblet cells^49–53^. The function of NLRP6 in the large intestine has been widely reported^54,55^, but the exact role of the NLRP6 inflammasome in small intestines remains elusive. Additionally, the role of the NLRP9 inflammasome in intestine is largely limited^56^. Here we found TRIM29 interacted NLRP6 and NLRP9b to suppress IL-18 production in small intestine IECs following enteric RNA virus infection.

Intraepithelial lymphocytes comprise a large T cell population located at the critical interface between the intestinal lumen and the core of the body^35,36^ and canprovide a first line of immunity in mice and humans while balancing tolerance and defense^37,38,39,40^. It’s reported that the newly recruited intraepithelial Ly6A^+^CCR9^+^CD4^+^ T cells in small intestines play protective role in host defense against infection of enteric viruses including murine norovirus and adenovirus^27^. Here, we demonstrate that TRIM29 deficiency recruits more intraepithelial Ly6A^+^CCR9^+^CD4^+^ T cells in small intestines to protect against enteric RNA viruses including rotavirus and EMCV. IL-18 is shown to be required for recruitment of intraepithelial Ly6A^+^CCR9^+^CD4^+^ T cells in small intestines during enteric virus infection. Additionally, the newly recruited intraepithelial CD4^+^ T cells are shown to play protective role in host defense against enteric adenovirus infection by producing protective IFN-γ and Granzyme B^27^. We also demonstrate significantly higher frequencies of intraepithelial IFN-γ-expressing CD4^+^ T cells and Granzyme B-expressing CD4^+^ T cells in small intestines from *Trim29*^IEC-KO^ adult mice than those from *Trim29*^fl/fl^ mice after rotavirus and EMCV infection. Our findings address how TRIM29 controls innate immune cytokines IL-18 production in IECs after enteric RNA virus infection and how the IL-18 production in enteric virus-induced epithelial cell impact the function and acquisition of innate-like properties by the intraepithelial Ly6A^+^CCR9^+^CD4^+^ T cells.

In conclusion, we demonstrate that IEC-specific TRIM29 deficiency controls infections by enteric RNA viruses by targeting NLRP6 and NLRP9b for degradation to enhance IFN-λ3 and IL-18 production in IECs and augment the recruitment of protective intraepithelial Ly6A^+^CCR9^+^CD4^+^ T cells in small intestines. Significantly, gastrointestinal symptoms and fecal shedding of SARS-CoV-2 RNA are frequently observed in COVID-19 patients and SARS-CoV-2 could efficiently infect human IECs ^6,7^. Thus, our insights into the role of TRIM29 in controlling enteric RNA virus-induced intestinal inflammation suggest potential therapeutic applications involving targeting TRIM29 to control gut diseases associated with SARS-CoV-2 infection.

## Materials and methods

### Mice

*Trim29*^—/—^ mice were obtained from the European Mouse Mutant Archive (EMMA)^28,29, 34^. *Trim29*^fl/fl^ mice were generated as previously described^43,57^. Briefly, *Trim29*-targeted mice were crossed with FLP-deleted mice (B6 ROSA26Flpo; Stock No: 012930, The Jackson Laboratory) to produce *Trim29*^fl/fl^ mice. The *Trim29*^fl/fl^ mice were further crossed with the *Villin-Cre* transgenic mice^58^ (Stock No: 021504, The Jackson Laboratory) that express Cre recombinase in villus and crypt epithelial cells of the small and large intestines to generate IEC-specific *Trim29*-knockout mice, *Trim29*^fl/fl^; *Villin*-Cre (*Trim29*^*IEC-KO*^). The Ifnar1 (type I IFN receptor) knockout (*Ifnar1*^*−/−*^; Stock No: 028288, The Jackson Laboratory) mice were crossed with *Trim29*^*fl/fl*^ and *Trim29*^*IEC-KO*^ mice to generate *Ifnar1*^*−/−*^*Trim29*^*fl/fl*^ and *Ifnar1*^*−/−*^ *Trim29*^*IEC-KO*^, respectively. All animals were on the C57BL/6 genetic background and maintained in the specific pathogen-free facility at Houston Methodist Research Institute in Houston, Texas. Animals were housed under the following conditions: temperatures of 68–72F, 30–70 % humidity, 10–15 fresh air exchanges hourly, and a 12:12 h light:dark cycle (lights were on from 07:00–19:00). Mice were housed in sterile individually-ventilated cages (Techniplast S.p.A., Buguggiate, Italy) containing autoclaved Bedo’Cobs 1/4″ bedding (The Andersons, Inc.), a sterile cotton nesting square or sterile crinkle nesting material, and received approximately 75 air changes hourly. Mice were housed at a density of up to five mice. All animal studies were ethically reviewed and approved by the Houston Methodist Animal Care Committee (IS00007198) and were carried out in accordance with the National Institutes of Health Guidelines for the Care and Use of Laboratory Animals.

### In vivo rotavirus infection

Rotavirus EW is a non-cell culture-adapted wild-type murine rotavirus strain. The virus titration of rotavirus was expressed as 50 % diarrhoea dose (DD50) defined as the highest dilution that causes diarrhoea in 50 % of suckling C57BL/6 mice ^59^. For rotavirus infection in mice, 8-day-old wild-type *Trim29*^+/+^ and *Trim29*^fl/fl^, *Trim29*^−/−^ and *Trim29*^*IEC-KO*^ mice were randomly divided into control mock and infection groups and were orally inoculated by gavage with 1 DD50 rotavirus in 50 μl PBS. The appearance of diarrhoea was monitored over time by changes in color and consistency of feces. On day 5 after infection, mice were euthanized and intestines were collected. Rotavirus titer in intestinal tissues was detected by quantitative RT-PCR (qRT-PCR) based on rotavirus gene 11 (NSP5) sequences. On day 1 after infection, mice without or with infection were euthanized and intestines were collected. The cytokines *Ifnl2/3,* and *Il18* in intestine tissues were determined by qRT-PCR. The cytokines IFN-λ and IL-18production in intestine homogenate was measured by ELISA. On day 4 after infection, mice without or with infection were euthanized and intestines were collected for flow cytometry analysis.

For diarrhoea experiment of rotavirus infection, diarrhoea was documented, and tissue samples were collected and measured in a double-blinded manner. The percentage and severity of diarrhoea among the littermates during the course of infection was recorded as previously described ^18,60^. In brief, diarrhea was scored on the basis of color, consistency and amount, and numbered as follows: 0 = normal; 1 = pasty; 2 = semi-liquid; 3 = liquid and consider score ≥ 2 as diarrhoea.

### In vivo EMCV infection

For encephalomyocarditis virus (EMCV) infection in mice, five-week-old wild-type *Trim29*^fl/fl^, *Trim29*^*IEC-KO*^, *Ifnar1*^*−/−*^*Trim29*^*fl/fl*^ and *Ifnar1*^*−/−*^ *Trim29*^*IEC-KO*^ mice were randomly divided into control mock and infection groups and were inoculated intragastrically with 1 × 10^8^ plaque-forming units (PFU) of EMCV^57,61^ (strain K, gift from Dr. Michael S. Diamond at Washington University in St. Louis) in PBS. For the survival experiments, mice were monitored daily for survival after EMCV infection. On day 1 post-infection, mice without or with infection were euthanized and the intestine tissue was excised and homogenized in PBS (1 ml PBS per 1 g tissue). The cytokine production in intestine homogenate was measured by ELISA. On day 4 post-infection, mice without or with infection were euthanized and the intestines were excised for flow cytometry analysis. On day 4 post-infection, mice were euthanized and the intestine tissue was excised for determining EMCV titer by standard plaque assays.

### In vivo EMCV infection with IL-18 and IFNLR1 inhibition

For encephalomyocarditis virus (EMCV) infection with IL-18 and IFNLR1 inhibition in mice, five-week-old wild-type *Trim29*^fl/fl^ and *Trim29*^*IEC-KO*^ mice were inoculated intragastrically with 1 × 10^8^ PFU of EMCV. For IL-18 and IFNLR1 (IFN-λ receptor) inhibitions, immediately following infection of EMCV, mice underwentintraperitoneal injections of PBS, IL-18 binding protein (IL-18BP, 50 μg/kg) or IFNLR1 blocking peptide (IFNLR1 BP, 50 μg/kg). These treatments were repeated once a day for two consecutive days. On day 4 after infection, mice with different treatments were euthanized and intestines were collected for flow cytometry analysis.

### Reagents

The high molecular weight poly I:C (poly I:C, Cat: tlrl-pic) was from InvivoGen. Lipofectamine 3000 was from Invitrogen. The proteasome inhibitor MG132 was from Sigma. The following antibodies were used for immunoprecipitation: anti-TRIM29 (1:100; A301–210A; Bethyl), anti-NLRP6 (1:100; SAB1302240; Sigma) and anti-NLRP9 (1:100; NBP2–24661; Novus Biologicals). The following antibodies were used for immunoblot analysis: anti-TRIM29 (1:1000; A301–210A; Bethyl), anti-TRIM29 (1:1000; sc-33151; H-300; Santa Cruz), anti-NLRP6 (1:100; SAB1302240; Sigma-Aldrich), anti-NLRP9 (1:100; NBP2–24661; Novus Biologicals), anti-caspase-1 (1:1000; AG-20B-0044; AdipoGen), anti-ubiquitin (1:1000; sc-8017; Santa Cruz), K63-specific anti-ubiquitin (1:1000; 05–1313; Millipore), K48-specific anti-ubiquitin (1:1000; 05–1307; Millipore), anti-GAPDH (1:10,000; clone GAPDH-71.1, G9295; Sigma), anti-HA (1:5000; clone HA-7, H6533; Sigma), anti-β-actin (1:20,000; clone AC-15, A3854; Sigma), anti-Myc (1:5000; 16–213; Sigma), and anti-Flag (1:5000; A8592; Sigma). Anti-HA and anti-Myc agarose beads were from Sigma. Lentiviral vectors for shRNA were from Dharmacon Inc. (Horizon Discovery Group company): human TRIM29 (clone TRCN0000016352). The human IFN-lambda 3 (IFN-λ3, D28B00), mouse IFN-lambda 3 (IFN-λ3, DIY1789B-05), human IL-18 (DY318–05) and mouse IL-18 (DY7625–05) ELISA kits were from R&D Systems. RNeasy Mini Kit (250, 74106) and QIAprep Spin Miniprep Kit (250, 27106) were from QIAGEN. The iScript cDNA Synthesis Kit (1708891) and iTaq Universal SYBR Green Supermix (1725125) were from Bio-Rad. Zombie Aqua Fixable Viability Kit (423102) was from BioLegend. Corning 354,234 Matrigel Matrix (CB-40234) was from Fisher Scientific. N-2 Supplement (17502048) and B-27 Supplement (17504044) were from Thermo Fisher. Recombinant Murine EGF (315–09), Recombinant Murine R-Spondin-1 (315–32) and Recombinant Murine Noggin (250–38) were from PeproTech. IWP-2 (HY-13912), Acetylcysteine (HY-B0215) and Laduviglusib (HY-10182) were from MCE.

### Cells culture and lentiviral infection

Human intestinal epithelial cells (IECs) line HT-29 was obtained from ATCC (ATCC HTB-38) and cultured in complete advanced DMEM/F12 medium. The HT-29 IECs were infected with a pLKO.1 lentiviral vector carrying a scrambled shRNA (sh-Ctrl, RHS6848, Horizon Discovery) or target gene sequences (sh-TRIM29, Horizon Discovery) as described in our previous studies ^28,29,62,63^. After 24 h of culture, cells were selected by the addition of puromycin (2 ng/ml) to the medium. For overexpression of TRIM29, the HT-29 IECs with sh-TRIM29 infection were transfected without or with HA-TRIM29 plasmid by Lipofectamine 3000 for 24 h followed by EMCV or Rotavirus infection at a multiplicity of infection (MOI) of 5 for quantitative RT-PCR analysis of viral replication. Cells were stimulated for 16 h with poly I:C (20 μg/ml) delivered by Lipofectamine 3000. The knockdown efficiency was detected with immunoblot analysis.

### Isolation of mouse intestinal epithelial cells

Isolation of mouse primary intestinal epithelial cells (IECs) was performed as described previously^64^. Briefly, 6-week-old C57BL/6 mouse intestines were opened longitudinally, washed in phosphate-buffered saline (PBS) and cut into 5-mm fragments. The epithelial integrity was disrupted by treatment with 1 mM dithiothreitol (DTT) on a shaker. Liberated IECs were collected and separated by Percoll gradient (Sigma Aldrich). Interface cells were collected and used as IECs. Purified IECs were cultured in high-glucose-formulated DMEM, supplemented with 10 % FBS, 4 mM glutamine, 20 mM Hepes, 1 mM sodium pyruvate, and 100 U/mL penicillin/streptomycin. The purity of isolated IEC was confirmed using FACS analysis with antibodies against IEC markers, PE-anti-E-Cadherin antibody (Catalog: 147304, BioLegend) and FITC-anti-Cytokeratin 18 antibody (Catalog: MA1–10326, ThermoFisher Scientific). Isolated IEC purity and survival rate were both > 95 %.

### Culture of primary IECs from mouse intestinal organoids

Six-week-old wild-type *Trim29*^+/+^ and *Trim29*^fl/fl^, *Trim29*^−/−^ and *Trim29*^*IEC-KO*^ mice were sacrificed, the small intestine excised, immersed in sterile cold PBS, cut lengthwise, and rinsed using sterile cold PBS to wash away the intestinal contents. The intestine was then cut into pieces (2–4 mm) and placed in a 50-mL centrifuge tube which contained sterile cold PBS, and a 2-ml pipette was used to wash the pieces via pipetting up and down repeatedly 15–20 times with sterile cold PBS. Then pieces were incubated for 15 min with 10 mL of 30 mM EDTA in PBS at room temperature. Subsequently, the reagent was removed, the intestinal sections were washed with PBS, and the supernatant fractions enriched in crypts were collected using a 70-μm cell strainer. These fractions were centrifuged at 400 × g for 5 min, following which the supernatant was removed. The precipitate was resuspended in 5 mL cold Dulbecco’s Modified Eagle Medium/Nutrient Mixture F-12 (DMEM/F12) solution (Gibco, Thermo Fisher Scientific), crypts were counted using an inverted microscope, and the required amount of liquid was aspirated into a centrifuge tube and centrifuged for 5 min at 400 × g. The precipitate was collected and resuspended in the same amount of organoid medium, including mouse epidermal growth factor (mEGF), Noggin, and R-spondin 1 (ENR medium), and Matrigel (354234, Corning, Fisher Scientific). The ENR medium contained DMEM/F12 media (Gibco), Primocin (100 μg/mL, InvivoGen), N2 Supplement (1X, Gibco), B27 Supplement (1X, Gibco), mouse epidermal growth factor (50 ng/mL, PeproTech), R-Spondin 1 (500 ng/mL, PeproTech), and murine Noggin (100 ng/mL, PeproTech). Next, the suspension (50 μL) was quickly inoculated on preheated cell culture dishes to form a dome-shaped gelatinous structure. The dishes were then placed in a 37 ^◦^C incubator for 20 min. After the Matrigel was solidified, ENR medium was added to the culture dishes for mouse intestinal organoids culture for 5 days to get primary IECs from mouse intestinal organoids. The primary IECs from mouse intestinal organoids were infected with enteric RNA viruses simian rotavirus SA-11 strain, which has been shown to replicate good in mouse intestinal organoids^31^, or EMCV at a multiplicity of infection (MOI) of 5. The supernatants were collected for ELISA analysis. The IECs were treated with proteasome inhibitor MG132 (5 μM) for 3 h and then lysed for immunoprecipitation and ubiquitination assays.

### Isolation of mouse intestine macrophages

Six-week-old wild-type *Trim29*^+/+^ and *Trim29*^−/−^ mice were sacrificed and the small intestines excised. Small intestines were washed three times with HBSS (Ca/Mg-free), and fat and Peyer’s patches were removed. Small intestines were then opened longitudinally, cut into 1-cm pieces, and incubated in HBSS containing 5 uM EDTA + 5 %FBS + 1 μM DTT. The tissues were then digested with Liberase (Sigma) for 30 mins at 37 ^◦^C on a rotor. The digested cell suspension was then passed through 100 μm cell strainers. Isolated intestinal cells were stained with FITC anti-mouse CD45 antibody (109806, BioLegend), Alexa Fluor 700 anti-mouse I-A/I-E (MHCII) antibody (107622, BioLegend), APC/Cyanine7 anti-mouse Ly-6G antibody (127624, BioLegend), Brilliant Violet 785 anti-mouse/human CD11b antibody (101243, BioLegend), PE/Cyanine7 anti-mouse F4/80 antibody (123114, BioLegend) and were subjected to flowcytometric sorting to purify intestinal macrophages^65^ (CD45^+^MHCII^+^ Ly6G^-^CD11b^+^F4/80^+^) using BD SORP FACS Aria-II machine. The mouse intestine macrophages were infected with enteric RNA virus Rotavirus at a MOI of 5. The supernatants were collected for ELISA analysis.

### Virus plaque titration

For EMCV infection in mice, viral titers in intestine from infected mice were determined by plaque assay on L929 cells ^66^. Weights of organs were measured before the assay, and PFU were calculated per mg of tissue. Briefly, tissue was homogenized in 800 μl of PBS. The homogenates were treated with chloroform (10 % final concentration), centrifuged briefly and serial dilutions of the aqueous supernatants were incubated on L929 cells at room temperature. After 1 h, the inoculum was removed, and cells were covered with 2 % agar solution with amphotericin-B. After six days, 2 % agar solution containing 2 % neutral red solution was added and plaques were visualized with neutral red on the second day ^29,67,68,69^.

### Histology

Intestines were removed from wild-type *Trim29*^fl/fl^ and *Trim29*^*IEC-KO*^ mice infected with EMCV. These removed intestines were washed using PBS before being fixed with 10 % formaldehyde for 24 h at room temperature. The tissues were embedded in paraffin and processed by standard techniques. Longitudinal 5-μm sections were stained with Haematoxylin & Eosin (H&E) and viewed with a digital inverted light microscope (EVOS, Thermo Fisher Scientific, Waltham, MA) as previously described ^28,29,70^. Histology score was assessed based on intestinal inflammation and tissue damage. Inflammation was assessed by the presence of infiltrating mononuclear cells, polymorphonuclear cells and lymphocytic cells (scores from 0 to 3, with 0 absent, 1 mild, 2 moderate, 3 severe). For the evaluation of tissue damage four scores were ascribed to crypt hyperplasia, epithelial injury and death of epithelial cells (0 absent, 1 mild, 2 moderate, 3 severe).

### Isolation of intraepithelial lymphocytes from intestine

Small intestine intraepithelial lymphocytes were isolated as previously described^18,27,71^. Briefly, small intestines were removed and placed in chilled Hanks′ Balanced Salt Solution (HBSS) media containing 5 % fetal calf serum (FCS). The intestines were carefully cleaned from the mesentery and flushed of fecal content. Intestines were opened longitudinally and then cut into 1 cm pieces. The intestinal tissue was transferred to a 50-ml tube containing 25 ml of preheated HBSS complemented with 2 % FCS and 1 mM dithiothreitol (DTT) and shaken at 200 rpm for 30 min at 37 ^◦^C. The tissue suspension was passed through a filter into 50-ml conical tubes and the cells were pelleted by centrifugation at 1200 rpm for 10 min at 4 ^◦^C. The cell pellet was resuspended in complete HBSS, layered over a discontinuous 40 % and 75 % Percoll gradient, and centrifuged with no brake at 2000 rpm for 20 min. Cells from the 40 % and 75 % Percoll interface were collected, washed and resuspended in complete RPMI 1640 media. These purified cells constituted the intraepithelial lymphocytes population.

### Flow cytometry

Mouse primary IECs were isolated from wild-type *Trim29*^fl/fl^ and *Trim29*^*IEC-KO*^ mice. The cells were then stained using a Zombie Aqua fixable viability kit (423102, BioLegend) followed by surface staining with APC/Cyanine7 anti-mouse CD45 antibody (30-F11, Biolegend), PE anti-mouse CD324 (E-Cadherin) antibody (DECMA-1, Biolegend), PE/Cyanine7 anti-mouse CD326 (EpCAM) antibody (G8.8, Biolegend) and their isotype matched control antibodies for their differentiation. The intraepithelial lymphocytes isolated from small intestines of wild-type *Trim29*^fl/fl^ and *Trim29*^*IEC-KO*^ mice were used for follow flow cytometry. For intracellular cytokine staining, cells were stimulated *in vitro* for 4 h with phorbol 12-myristate 13-acetate (50 ng/ml) and ionomycin (550 ng/ml; Sigma-Aldrich) in the presence of GolgiStop (BD Biosciences) before staining. Viability was determined by LIVE/DEAD staining using a Zombie Aqua fixable viability kit (423102, BioLegend). For intracellular cytokine staining, cells were resuspended in fixation and permeabilization solution (00–5523–00, eBioscience). Cells were blocked with anti-CD16/CD32 antibody (101320, BioLegend) and stained with fluorochrome-conjugated monoclonal antibodies. The following antibodies were used to analyze the composition of Ly6A^+^CCR9^+^CD4^+^ T cells, IFN-γ-producing CD4^+^ T cells and Granzyme B-producing CD4^+^ T cells: APC/Cyanine7 anti-mouse CD45 antibody (103116, BioLegend), FITC anti-mouse CD3 antibody (100204, BioLegend), PE/Cyanine7 anti-mouse CD4 antibody (100528, BioLegend), PerCP/Cyanine5.5 anti-mouse CD8a antibody (100734, BioLegend), Alexa Fluor 647 anti-mouse CD199 (CCR9) antibody (128708, BioLegend), PE anti-mouse Ly-6A/E (Sca-1) antibody (160906, BioLegend), APC anti-mouse IFN-γ antibody (505810, BioLegend) and PE anti-human/mouse Granzyme B antibody (372208, BioLegend). Flow cytometry data were acquired on an LSR-II flow cytometer (Beckton Dickinson) and analyzed using FlowJo v10 software (Tree Start) as previously described ^18,28^.

### In vitro immunoprecipitation and immunoblot analysis

For the endogenous immunoprecipitation interaction assay, primary IECs from mouse intestinal organoids were infected with or without Rotavirus or EMCV at MOI of 5 for 4 h and then treated with proteasome inhibitor MG132 (5 μM) for 3 h and finally lysed with lysis buffer (50 mM Tris-Cl [pH7.5], 1 mM EDTA, 150 mM NaCl, 1.0 % NP-40) containing protease inhibitor cocktail (ThermoFisher Scientific). The cell lysates were incubated with anti-TRIM29 antibody and protein A/G agarose beads for immunoblot analysis. For the preparation of purified TRIM29, NLRP6 and NLRP9b, HEK293T cells were transfected with expression plasmids encoding full-length or truncated versions of HA-tagged TRIM29, NLRP6 and NLRP9b or full-length Myc tagged TRIM29, NLRP6 and NLRP9b constructed in pCMV-HA vector (Catalog: 635690, Clontech) or pCMV-Myc vector (Catalog: 635689, Clontech) as described in our previous study ^72^. Lysates were prepared from the transfected cells, followed by incubation with anti-HA or anti-Myc beads. Proteins were eluted from the beads after beads were washed six times with PBS. For precipitation with anti-Myc beads, purified HA-tagged full-length or truncations of TRIM29, NLRP6 or NLRP9b were incubated for 2 h with purified Myc-tagged NLRP6, NLRP9b or TRIM29. Beads were added; after 2 h of incubation, the bound complexes were pelleted by centrifugation. Proteins and beads were analyzed by immunoblot analysis. HT-29 IECs or mouse primary IECs were washed twice with phosphate-buffered saline (PBS) on ice and lysed in NP-40 lysis buffer with complete protease inhibitor for immunoblot analysis. For immunoblot analysis, all protein samples were dissolved in SDS sample buffer and resolved by 10–15 % SDS-PAGE. After electrophoresis, separated proteins were transferred onto polyvinylidenedifluoride (PVDF) membrane. The membrane was then blocked with 5 % nonfat milk. After incubation with specific primary antibody, horseradish peroxidase-conjugated secondary antibody was applied. The positive immune reactive signal was detected by an enhanced chemiluminescence system (ThermoFisher Scientific) as previously described ^28,67,68,69,73,74^.

### NLRP6 and NLRP9b inflammasome reconstitution in HEK293T cells

HEK293T cells were plated in six-well microplates and incubated overnight. The cells were transfected with plasmids including HA-pro-IL-18 (MG50073-CY, SinoBiological, 1000 ng/well), Myc-ASC (Cat: 73952, Addgene, 200 ng/well), Flag-Caspase-1 (Cat: 21142, Addgene, 200 ng/well), with or without Myc-NLRP6 (500 ng/well) or Myc-NLRP9b (500 ng/well), HA-TRIM29 or HA-Vector (100 or 500 ng/well) using Lipofectamine 3000. Cells were collected 24 h after transfection and lysed in NP-40 buffer with complete protease inhibitors. IL-18 maturation was assessed by immunoblot analysis^18,70^.

### Quantitative RT-PCR

RNA was isolated using the RNeasy Kit (Qiagen) according to the manufacturer’s instructions. The isolated RNA was used to synthesize cDNA with the iScript cDNA Synthesis Kit (Bio-Rad). iTaq SYBR Green Supermix with ROX (Bio-Rad) was used for quantitative RT-PCR (qRT-PCR) ^34,75–77^. PCRs were performed in triplicate. Primer sequences used for qRT-PCR are shown in Table. S1.

### Quantification and statistical analysis

A two-tailed unpaired Student’s *t* test was used for statistical analysis with Microsoft Excel and GraphPad Prism Software. Statistical significance was determined by the probability (*P*) values, denoted by asterisks, which were set at < 0.05 (*), <0.01 (**), and < 0.001 (***), respectively. Any data that failed to reach statistical significance with *P* values greater than 0.05 were represented as “not significant” (NS). TheGehan-Breslow-Wilcoxon test was used for survival analysis.

## Supplementary Material

Supplement

Appendix A. Supplementary data

Supplementary data to this article can be found online at https://doi.org/10.1016/j.mucimm.2024.10.004.
