# Supplementary material for: TRIM29 controls enteric RNA virus-induced intestinal inflammation by targeting NLRP6 and NLRP9b signaling pathways
Source: Mucosal Immunol. Author manuscript; Available in PMC 2025 Jul 1. (PMC12210021; doi:10.1016/j.mucimm.2024.10.004)
Supplement: Supplement [file NIHMS2071111-supplement-Supplement.pdf]

**TRIM29 controls enteric RNA virus-induced intestinal inflammation by targeting NLRP6 and NLRP9b signaling pathways**

Junying Wang[a,1], Ling Wang[a,b,1], Wenting Lu[a,1], Naser Farhataziz[a], Anastasia Gonzalez[a], Junji Xing[a,c,d,*], Zhiqiang Zhang[a,d,*]

[a]Immunobiology and Transplant Science Center, Department of Surgery, Houston Methodist Academic Institute, Houston Methodist, Houston, TX 77030, USA

[b]Department of Obstetrics and Gynecology, The Second Hospital of Jilin University, Changchun, 130021, China

[c]Department of Cardiovascular Sciences, Houston Methodist Academic Institute, Houston Methodist, Houston, TX 77030, USA

[d]Department of Surgery, Weill Cornell Medicine, Cornell University, New York, NY 10065, USA

[1]These authors contributed equally

[*]Correspondence: jxing@houstonmethodist.org (J. Xing), zzhang@houstonmethodist.org (Z. Zhang)

This document includes Supplementary Figures 1-8 and Supplementary Table 1.

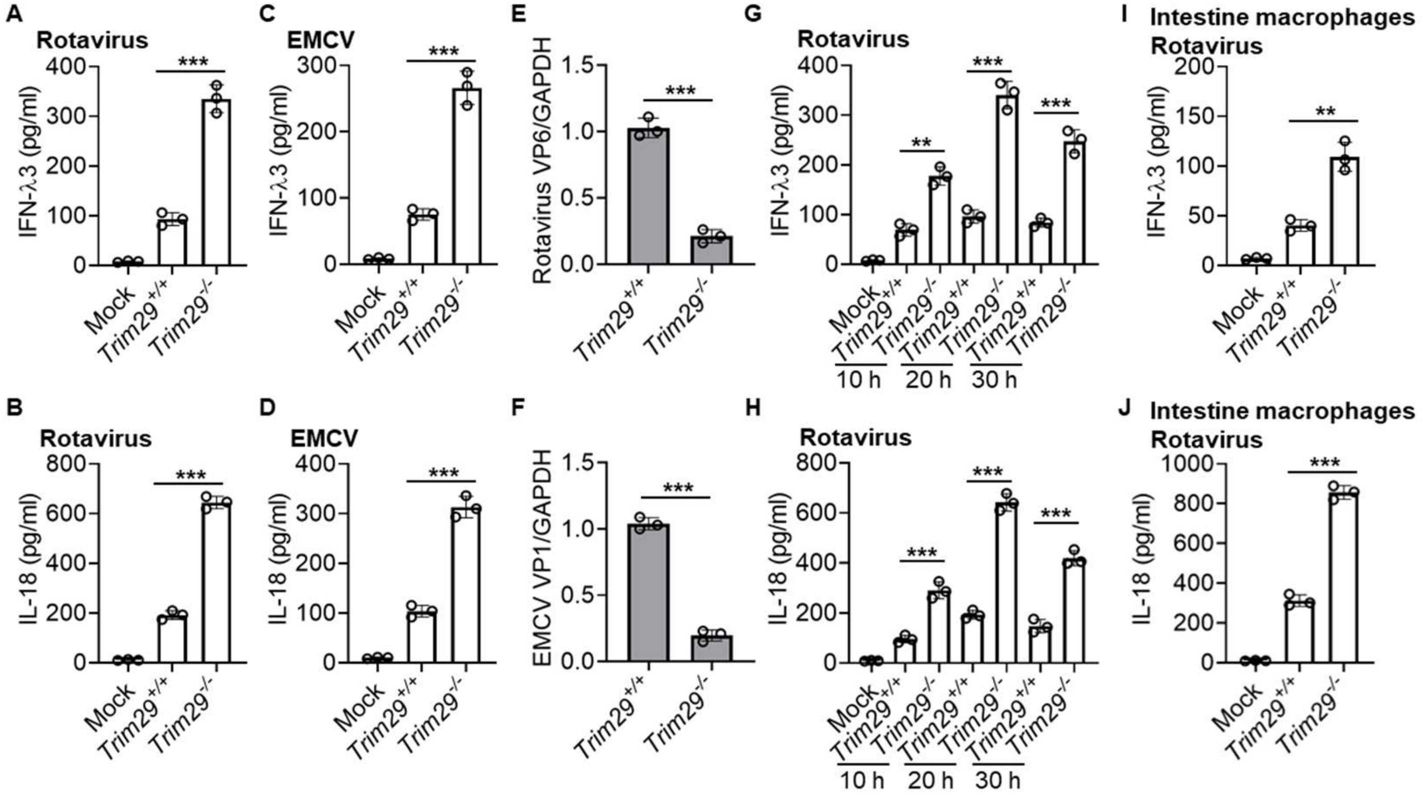


**Supplementary Figure 1. Knockout of TRIM29 produces more IFN-λ3 and IL-18 to restrict viral replication in primary IECs from mouse intestinal organoids and intestine macrophages after infection with Rotavirus and EMCV.**

(**A-D**) ELISA of IFN-λ3 (**A, C**) and IL-18 (**B, D**) production in primary IECs from mouse intestinal organoids of wild-type *Trim29*$^{+/+}$ and *Trim29*$^{-/-}$ mice after a 20 h infection with enteric RNA viruses including simian rotavirus SA-11 strain (**A-B**) and EMCV strain K (**C-D**) at a MOI of 5. Mock, cells without virus infection. (**E-F**) Quantification of expression of rotavirus VP6 gene (**E**) and EMCV VP1 gene (**F**) relative to GAPDH in primary IECs from mouse intestinal organoids of wild-type *Trim29*$^{+/+}$ and *Trim29*$^{-/-}$ mice infected by rotavirus (**E**) or EMCV (**F**) as in **A,C**. (**G-H**) ELISA of IFN-λ3 (**G**) and IL-18 (**H**) production in primary IECs from mouse intestinal organoids of wild-type *Trim29*$^{+/+}$ and *Trim29*$^{-/-}$ mice after infection without (Mock) or with simian rotavirus SA-11 strain at a MOI of 5 for 10 h, 20 h or 30 h. (**I-J**) ELISA of IFN-λ3 (**I**) and IL-18 (**J**) production in mouse intestine macrophages from wild-type *Trim29*$^{+/+}$ and *Trim29*$^{-/-}$ mice after a 20 h infection with simian rotavirus SA-11 strain at a MOI of 5. Data are represented as mean ± SD. ***P*<0.01, ****P*<0.001 (unpaired t test). Data are representative of three technical replicates.

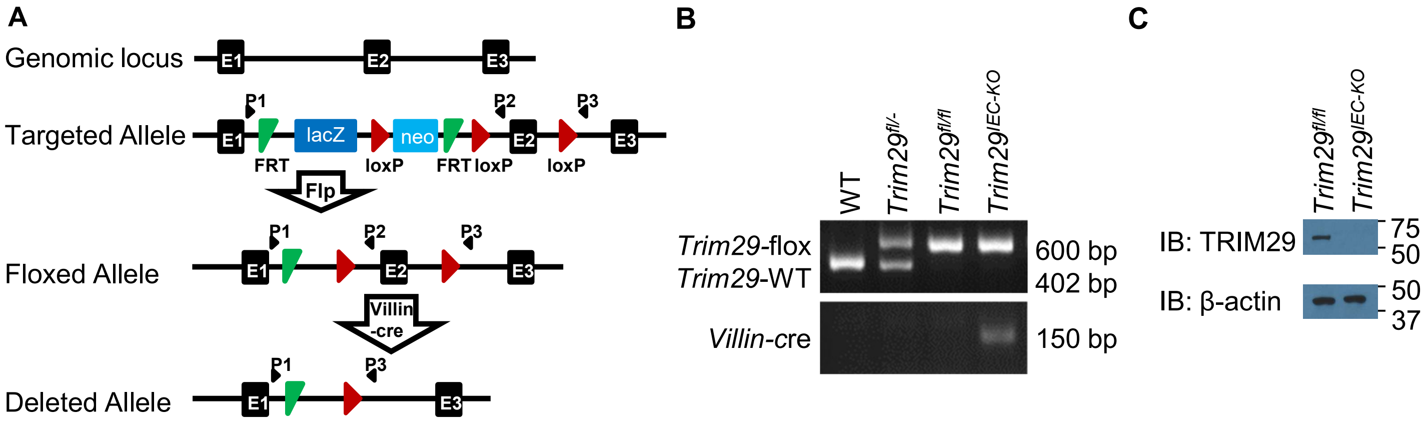


**Supplementary Figure 2. Trim29 gene targeting.**

(**A**) Schematic picture of Trim29 gene targeting using an FRT-LoxP vector, showing the exons 1 to 3 of Trim29 gene. Targeted mice were crossed with FRT deleter (Rosa26-FLPe) mice to generate *Trim29*-floxed (*Trim29*$^{fl/fl}$) mice, which were further crossed with *Villin*-Cre transgenic mice to generate IEC-specific *Trim29*-knockout mice, *Trim29*$^{fl/fl}$; *Villin*-Cre (*Trim29*$^{IEC\text{-}KO}$).

(**B**) Genotyping PCR to amplify the *Trim29-flox* (using P1/P2 primer pair) and WT (using P1/P2 primer pair) alleles (top), or the *Villin*-Cre (using 16775/oIMR9074 primer pair) alleles (bottom).

(**C**) Immunoblot (IB) of TRIM29 in mouse primary IECs from wild-type *Trim29*$^{fl/fl}$ and *Trim29*$^{IEC\text{-}KO}$ mice. The position of protein markers (shown in kDa) is indicated on the right.

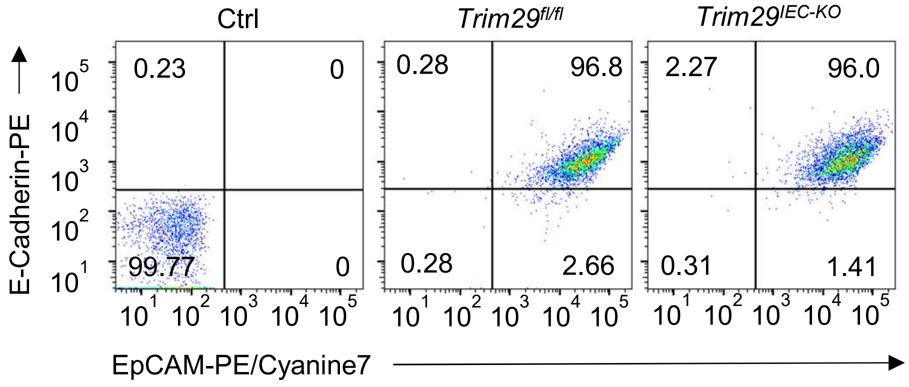


**Supplementary Figure 3. TRIM29 does not affect expression of differentiation markers EpCAM and E-Cadherin in mouse IECs.**

Flow cytometry analyzing the expression of differentiation markers EpCAM and E-Cadherin in the mouse primary IECs isolated from wild-type *Trim29*$^{fl/fl}$ and *Trim29*$^{IEC\text{-}KO}$ mice using isotype control antibodies (Control, Ctrl), EpCAM-PE/Cyanine7 and E-Cadherin-PE antibodies. Flow cytometry data were acquired on an LSR-II flow cytometer (Beckton Dickinson) and analyzed using FlowJo v10 software (Tree Star). Data are representative of three technical replicates.

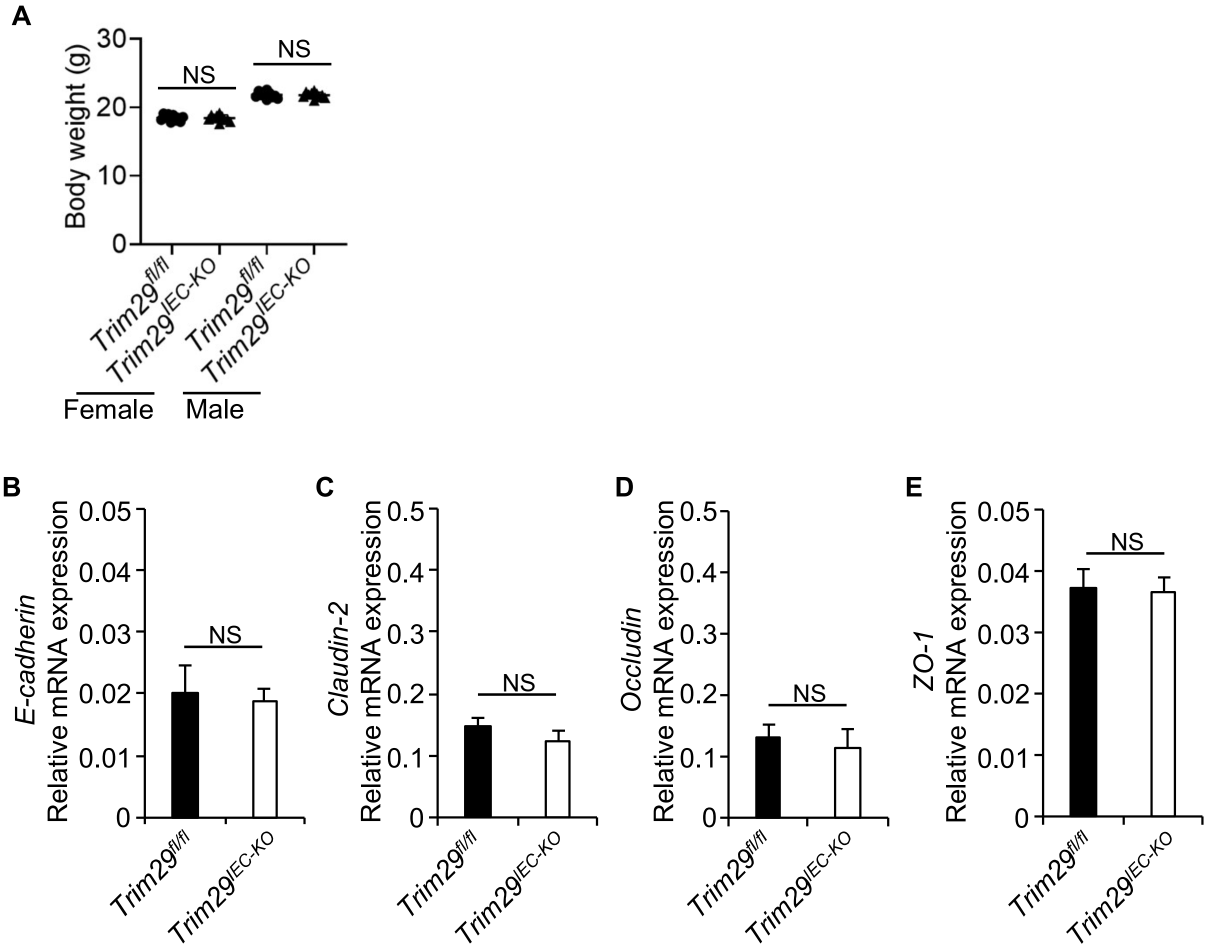


**Supplementary Figure 4. IEC-specific TRIM29 ablation does not affect body weight and expression of epithelial tight junction proteins.**

(**A**) Body weight analysis of 6-week-old *Trim29*$^{fl/fl}$ and *Trim29*$^{IEC\text{-}KO}$ female and male mice (n=10). (**B-E**) The qRT-PCR analysis of the expression of tight junction-related genes E-cadherin (**B**), Claudin-2 (**C**), Occludin (**D**), and Zonula occludens-1 (ZO-1, **E**) in the mouse primary IECs isolated from wild-type *Trim29*$^{fl/fl}$ and *Trim29*$^{IEC\text{-}KO}$ mice. mRNA, messenger RNA. NS, not significant. Data are representative of three technical replicates.

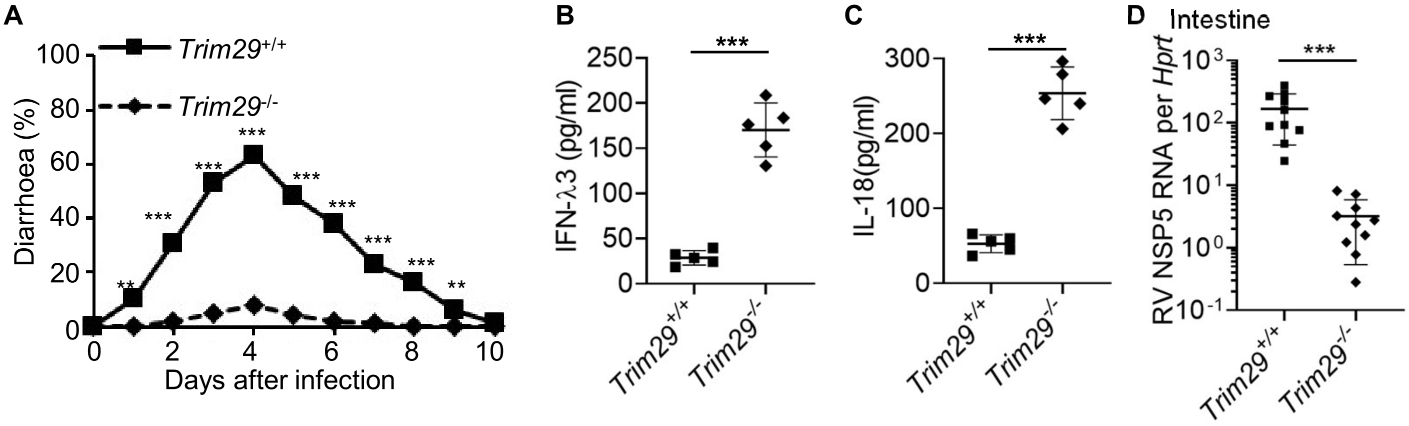


**Supplementary Figure 5. TRIM29 knockout controls intestinal inflammation induced by enteric rotavirus infection in suckling mice *in vivo*.**

(**A**) Diarrhoea duration and percentage of mice with diarrhoea (score ≥2) from 8-day-old wild-type *Trim29*$^{+/+}$ and *Trim29*$^{-/-}$ suckling mice (n=10 per strain) orally inoculated by gavage with 1 DD50 rotavirus EW strain. (**B,C**) The wild-type *Trim29*$^{+/+}$ and *Trim29*$^{-/-}$ suckling mice (n=5 per strain) were orally inoculated by gavage with 1 DD50 rotavirus EW strain. At day 1 post-inoculation, mice were euthanized, and intestine tissues were excised and homogenized in PBS for detection of IFN-λ3 (**B**) and IL-18 (**C**) by ELISA. (**D**) The wild-type *Trim29*$^{+/+}$ and *Trim29*$^{-/-}$ suckling mice (n=10 per strain) were orally inoculated by gavage with 1 DD50 rotavirus EW strain. At day 5 post-inoculation, mice were euthanized, and intestine tissues were collected for qRT-PCR detection of rotavirus levels. Mock, mouse without rotavirus infection. Data are represented as mean ± SD. **P<0.01 and ***P<0.001 (unpaired t test). Data are representative of three experiments.

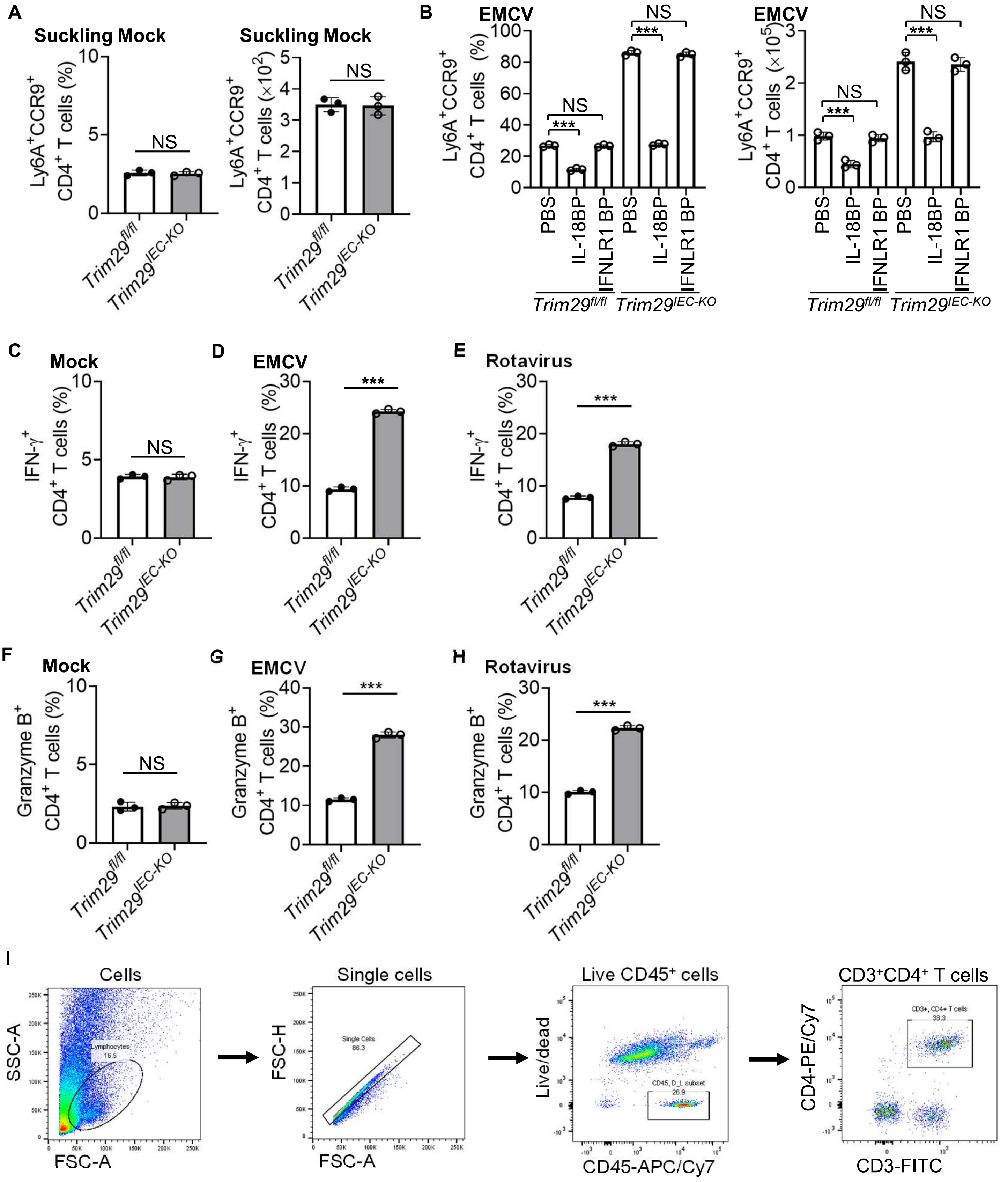

A
Suckling Mock
Ly6A+CCR9+ CD4+ T cells (%)
Ly6A+CCR9+ CD4+ T cells (×10^2)
NS
Trim29fl/fl
Trim29IEC-KO
B
EMCV
Ly6A+CCR9+ CD4+ T cells (%)
Ly6A+CCR9+ CD4+ T cells (×10^5)
NS
***
PBS
IL-18BP
IFNLR1 BP
C
Mock
IFN-γ+ CD4+ T cells (%)
D
EMCV
E
Rotavirus
F
Mock
Granzyme B+ CD4+ T cells (%)
G
EMCV
H
Rotavirus
I
Cells
SSC-A
FSC-A
Lymphocytes
16.5
Single cells
FSC-H
Single Cells
86.3
Live CD45+ cells
Live/dead
CD45-APC/Cy7
CD45, D_L subset
26.9
CD3+CD4+ T cells
CD4-PE/Cy7
CD3-FITC
CD3+, CD4+ T cells
38.3

**Supplementary Figure 6. Schematic diagram showing the gating strategy for $CD3^+CD4^+$ T cells in intraepithelial leukocytes of intestine.**

(**A**) Flow cytometry percent and absolute cell number quantification analysis of mouse intraepithelial $Ly6A^+CCR9^+CD4^+$ T in small intestine intraepithelial lymphocytes from both *Trim29*$^{fl/fl}$ and *Trim29*$^{IEC\text{-}KO}$ suckling mice without infection (Mock) using Ly6A-PE and CCR9-AF647 antibodies. (**B**) Flow cytometry percent and absolute cell number quantification analysis of mouse intraepithelial $Ly6A^+CCR9^+CD4^+$ T in small intestine intraepithelial lymphocytes from both *Trim29*$^{fl/fl}$ and *Trim29*$^{IEC\text{-}KO}$ adult mice infected with EMCV followed by intraperitoneal injections of PBS, IL-18 binding protein (IL-18BP, 50 μg/kg) or IFNLR1 blocking peptide (IFNLR1 BP, 50 μg/kg) once a day for two consecutive days using Ly6A-PE and CCR9-AF647 antibodies. (**C-H**) Quantification analysis of mouse intraepithelial IFN-γ producing $CD4^+$ T cells (IFN-$\gamma^+$ $CD4^+$ T cells, **C-E**) or Granzyme B producing $CD4^+$ T cells (Granzyme $B^+$ $CD4^+$ T cells, **F-H**) in small intestine intraepithelial lymphocytes from both *Trim29*$^{fl/fl}$ and *Trim29*$^{IEC\text{-}KO}$ adult mice infected without (Mock, **C, F**) or with EMCV (**D, G**), or both *Trim29*$^{fl/fl}$ and *Trim29*$^{IEC\text{-}KO}$ suckling mice infected with Rotavirus (**E, H**) for 3 days using CD4-PE/Cy7 and IFN-γ-APC or Granzyme B-PE antibodies. (**I**) Representative FACS plots showing the gating strategy for analyzing $CD3^+CD4^+$ T cells in small intestine intraepithelial leukocytes from wild-type *Trim29*$^{fl/fl}$ and *Trim29*$^{IEC\text{-}KO}$ mice. Flow cytometry data were acquired on an LSR-II flow cytometer (Beckton Dickinson) and analyzed using FlowJo v10 software (Tree Star). Data are shown as the mean ± SD. NS, $P>0.05$, and ***$P<0.001$ (unpaired t test). Data are representative of three experiments.

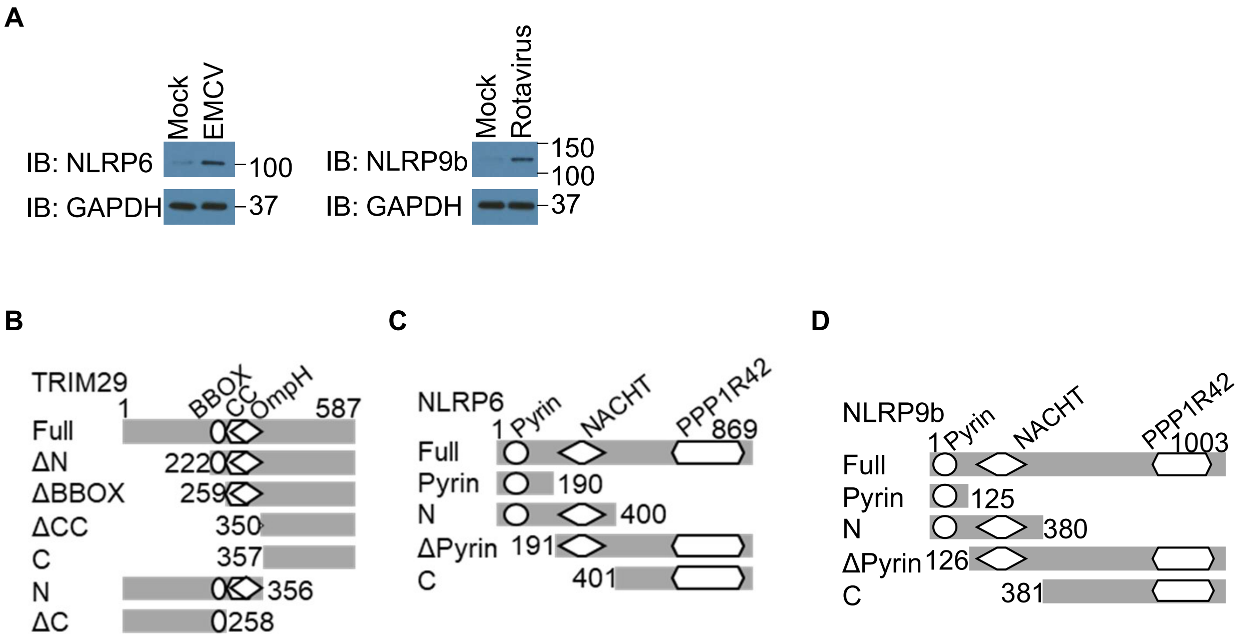


**Supplementary Figure 7. Expression of NLRP6 and NLRP9b in human HT-29 IECs and schematic diagram showing full-length and serial truncations of TRIM29, NLRP6 and NLRP9b.**

(**A**) Immunoblot (IB) showing the expression of NLRP6 and NLRP9b in human HT-29 IECs infected without (Mock) or with EMCV and rotavirus, respectively. (**B**) Schematic diagram showing full-length (Full) TRIM29 and serial truncations of TRIM29 with deletion (Δ) of various domains (left margin); numbers at ends indicate amino acid positions (top). BBOX, B-box domain; CC, Coiled-coil domain; OmpH, outer membrane protein H domain. (**C**) Schematic diagram showing Full NLRP6 and serial truncations of NLRP6 with deletion (Δ) of various domains (left margin); numbers at ends indicate amino acid positions (top). Pyrin, Pyrin death domain; NACHT, NTPase domain; PPP1R42, protein phosphatase 1 regulatory subunit 42 domain. (**D**) Schematic diagram showing Full NLRP9b and serial truncations of NLRP9b with deletion (Δ) of various domains (left margin); numbers at ends indicate amino acid positions (top).

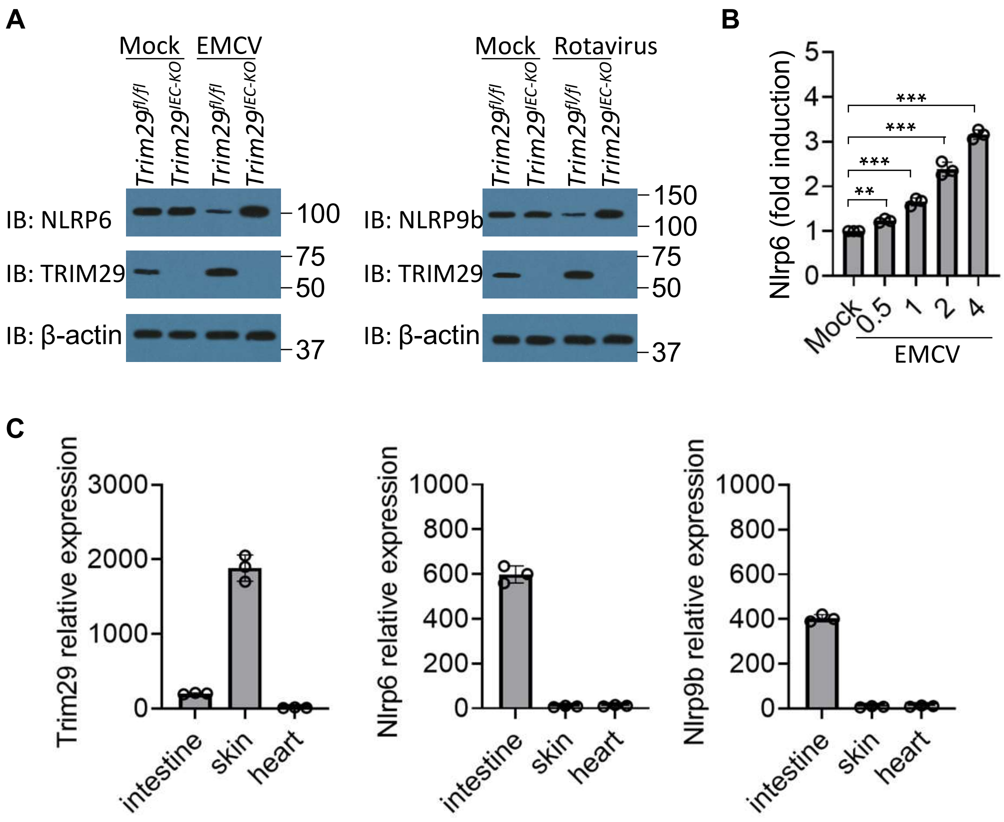


**Supplementary Figure 8. Expression regulation of NLRP6 and NLRP9b by TRIM29 in IECs of virus infected intestines *in vivo* and IECs from mouse intestine organoids and different tissues.**

(**A**) Immunoblot (IB) analysis of NLRP6, NLRP9b and TRIM29 in primary IECs from small intestines of *Trim29*$^{fl/fl}$ and *Trim29*$^{IEC\text{-}KO}$ mice infected without (Mock) or with EMCV or Rotavirus for 2 days. (**B**) RT-qPCR mRNA analysis of Nlrp6 and Nlrp9b in primary IECs from mouse intestinal organoids of wild-type *Trim29*$^{fl/fl}$ mice infected without (Mock) or with EMCV or Rotavirus at MOI of 5 for 0.5h, 1h, 2h or 4h. (**C**) RT-qPCR mRNA analysis of Trim29, Nlrp6 and Nlrp9b in intestine, skin and heart of wild-type *Trim29*$^{fl/fl}$ mice.

**Supplementary Table 1. Primers for qRT-PCR and genotype PCR used in this study.**

| Gene | Sequence |
|---|---|
| qRT-PCR | |
| Human *Gapdh* | F: 5'- GGAGCGAGATCCCTCCAAAAT -3' |
| | R: 5'- GGCTGTTGTCATACTTCTCATGG -3' |
| Mouse *Ifnl2/3* | F: 5'- AGTGGAAGCAAAGGATTG -3' |
| | R: 5'- GAGATGAGGTGGGAACTG -3' |
| Mouse *Il18* | F: 5'- GCCTCAAACCTTCCAAATCA -3' |
| | R: 5'- TGGATCCATTTCCTCAAAGG -3' |
| Mouse *Gapdh* | F: 5'- AGGTCGGTGTGAACGGATTTG -3' |
| | R: 5'- TGTAGACCATGTAGTTGAGGTCA -3' |
| Mouse *Hprt* | F: 5'- CACAGGACTAGAACACCTGC -3' |
| | R: 5'- GCTGGTGAAAAGGACCTCT -3' |
| Mouse *E-cadherin* | F: 5'- CACCTGGAGAGAGGCCATGT -3' |
| | R: 5'- TGGGAAACATGAGCAGCTCT-3' |
| Mouse *Claudin-2* | F: 5'- TATGTTGGTGCCAGCATTGT-3' |
| | R: 5'- TCATGCCCACCACAGAGATA-3' |
| Mouse *Occludin* | F: 5'- CCTCCAATGGCAAAGTGAAT-3' |
| | R: 5'- CTCCCCACCTGTCGTGTAGT-3' |
| Mouse *ZO-1* | F: 5'- CCACCTCTGTCCAGCTCTTC -3' |
| | R: 5'- CACCGGAGTGATGGTTTTCT -3' |
| Mouse *β-actin* | F: 5'- CGTGAAAAGATGACCCAGATCA -3' |
| | R: 5'- CACAGCCTGGATGGCTACGT -3' |
| Mouse *Trim29* | F: 5'- ACTCCTCCTTCTCCCTGAAA -3' |
| | R: 5'- GACATAGAATGGCCGGTAGTG -3' |
| Mouse *Nlrp6* | F: 5'- AGCTGAGAACGCTGTGTCG -3' |
| | R: 5'- AACTTGGGAACCCCGAAGC -3' |
| Mouse *Nlrp9b* | F: 5'- CGAAAATCGAGAATTCTTCC -3' |
| | R: 5'- ACCTGTAGAAACAGGCTTAAC -3' |
| Rotavirus VP6 | F: 5'- GCACAGCCATTCGAACATCATGC -3' |
| | R: 5'- TGCATCGGCGAGTACAGACTC -3' |
| EMCV VP1 | F: 5'- GGAGTTGAGAATGCTGAGAG -3' |
| | R: 5'- TCCAGGGTTTCTTACTCCTTGG -3' |
| Trim29 genotype PCR | |
| P1 | 5'-TCCCATCTTGCCTACACTGC -3' |
| P2 | 5'-TCTGCTGGGCTCTGTGCTAC -3' |
| P3 | 5'-TCGTGGTATCGTTATGCGCC -3' |
| Villin-Cre genotype PCR | |
| Common primer 16775 | 5'-GCCTTCTCCTCTAGGCTCGT -3' |
| WT primer 16776 | 5'-TATAGGGCAGAGCTGGAGGA -3' |
| Mutant primer oIMR9074 | 5'-AGGCAAATTTTGGTGTACGG -3' |
